# Switching between Limiting Charge Extraction Regimes in an Illuminated Semiconductor–Metal–Organic Framework Junction

**DOI:** 10.1021/jacs.5c05700

**Published:** 2025-06-10

**Authors:** Amol Kumar, Jingguo Li, Anna M. Beiler, Sascha Ott

**Affiliations:** 1 Department of Chemistry—Ångström Laboratory, 8097 Uppsala University, P.O. Box 523, 75237 Uppsala, Sweden; 2 Department of Environmental Science and Engineering, 594410 University of Science and Technology of China, Hefei 230026, China

## Abstract

Surface modification is an effective method to realize high performance photoelectrodes. While current investigations mostly aim to leverage surface layers for improved charge carrier kinetics during charge separation, interfacial charge transfer, and decreased recombination, carrier transport within the surface layer is largely unattended. Herein, we explore this charge transport process on a model photocathode consisting of p-Si and GaP semiconductors (SCs) that are coated with a redox-active Zn-NDI (NDI = naphthalene diimide bis-pyrazolate) metal–organic framework (MOF) surface layer. The MOF layer is able to accept photogenerated electrons and support a large photovoltage of the underlying SC. In addition to well-established carrier generation and interfacial transfer processes that are frequently considered to control photocurrents, experimental photoelectrochemical data of the MOF@SC electrodes expose limitations that arise from electron transport in the surface layer coating. The transport-limited regime becomes relevant when the illumination intensity is gradually increased and is sensitive to the nature of the underlying semiconductor as well as the electrolyte. The phenomenon reported in this work is likely present in other surface-modified photoelectrodes with thick cocatalysts or redox-active polymer coatings but can easily be overlooked. In the MOF@SC construct, the transition between different limiting regimes can be visualized owing to the well-behaved cation-coupled photoelectron hopping transport in the MOF layer. These findings support the design and realization of efficient photoelectrodes.

## Graphical abstract

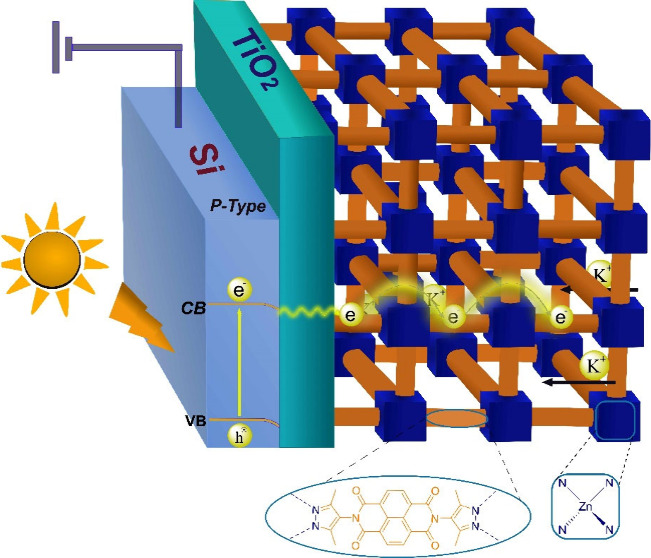

## Introduction

Photoelectrochemical (PEC) systems have emerged as one of the most promising yet challenging platforms for sustainable energy conversion.^1,2^ Among various strategies explored to enhance PEC efficiency, surface modification stands out for its ease of implementation and its ability to minimize charge carrier losses. So far, most research on surface-modified photoelectrodes targets means to enhance charge separation, facilitate interfacial charge transfer, and minimize charge recombination, as these factors are widely considered critical for improving PEC performance.^3^ However, the role of charge transport within surface modification layers (cocatalysts, passivation, or stabilization layers, etc.) has been largely overlooked, leaving a gap in understanding factors that govern overall PEC efficiency.^4–8^ In fact, transport limitations have been theoretically predicted but to the best of our knowledge not experimentally demonstrated.^9,10^ To bridge this gap, it is essential to employ model systems with well-defined structural and electrochemical properties so that the charge transport characteristics in the surface layer can selectively be comprehended. Metal–organic frameworks (MOFs), in particular those that are electrically conducting by redox hopping, constitute such materials and can be used to increase our understanding of limiting regimes for charge extraction in illuminated surface-coated semiconductors (SCs). At the same time, these types of MOFs themselves are promising for renewable energy technologies due to their structural and functional tunability, exposed active sites, and structural stability.^11–13^

To realize the full potential of the PEC, it is crucial to understand the influence of each elementary step. For the studies reported herein, SCs were coated with a well-defined MOF surface layer, interfaced by a ∼1 nm thin tunneling barrier of TiO_2_, the purpose of which is to protect the underlying SC from corrosion and to provide a robust MOF attachment. The MOF of choice is Zn-NDI (NDI = naphthalene diimide bis-pyrazolate, Figure 1a), which is a redox-conducting MOF that is electrically conducting by means of individual electron transfer steps between NDI linkers of different oxidation state. The Zn-NDI layer in the Zn-NDI|TiO_2_|SC photocathode does not absorb in the visible part of the spectrum itself, and light is exclusively absorbed by the SC. The efficiency of the latter process is an intrinsic parameter of the SC, and it depends on its band gap and absorption coefficient (Scheme 1a). Under external applied potentials, band bending of the underlying semiconductor increases, slowing down charge recombination and providing drift of photogenerated minority charge carriers toward the solid–electrolyte interface.^14^ Charge carrier lifetime and mobility determine the number of free carriers that reach the interface, which in turn determines the photovoltage (*V*_ph_) obtained by the system (Scheme 1b).^15^ At the interface, accumulated electrons tunnel through the thin TiO_2_ layer and get injected into the redox-active NDI linkers,^16,17^ a process that is in competition with recombination of the photoelectrons at the interface (Scheme 1c). A large surface concentration of redox-active units such as the NDI linkers in Zn-NDI (ca. 1 M) can be used to extract minority carriers from the illuminated SC. These electrons have to be channeled away through the MOF, and it is thus important to consider charge transport within the porous framework. In such cases, electron diffusion–migration in the surface layer^18^ constitutes an additional term to consider when evaluating the overall PEC performance of such a SC system with a surface coating (Scheme 1d).

**Scheme 1:**
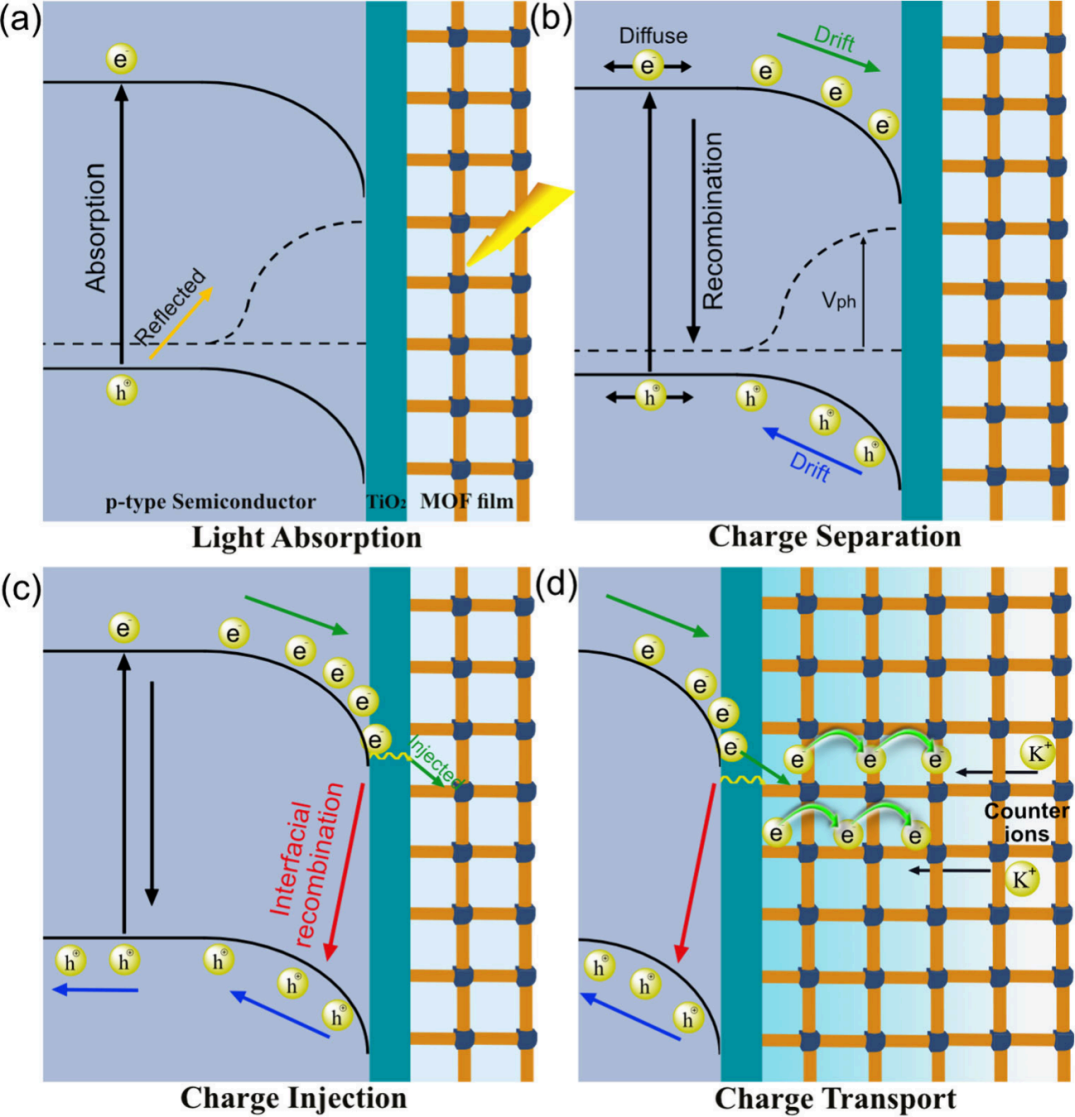
Illustration of the Illuminated Zn-NDI|TiO_2_|Si Photocathode, Showing the Limitation of Each Elementary Step^a^

a (a) Light absorption is an intrinsic parameter that depends on the band gap and absorption coefficient of the p-type SC. (b) Band bending encourages the drift of photo-electrons and holes towards and away from the SC surface respectively, with charge carrier lifetime and the absorption depth of the SC determining the number of charge-separated pairs reaching the interface. (c) At the interface, electrons tunnel through the thin TiO_2_ layer (∼1 nm) and are injected into the MOF film or recombine at the interface. (d) Injected electrons diffuse through the MOF thin film by hopping transport, coupled to the translocation of charge balancing counterions, and experimentally quantified by the apparent electron diffusion coefficient (*D*_e_^app^).

**Figure 1:**
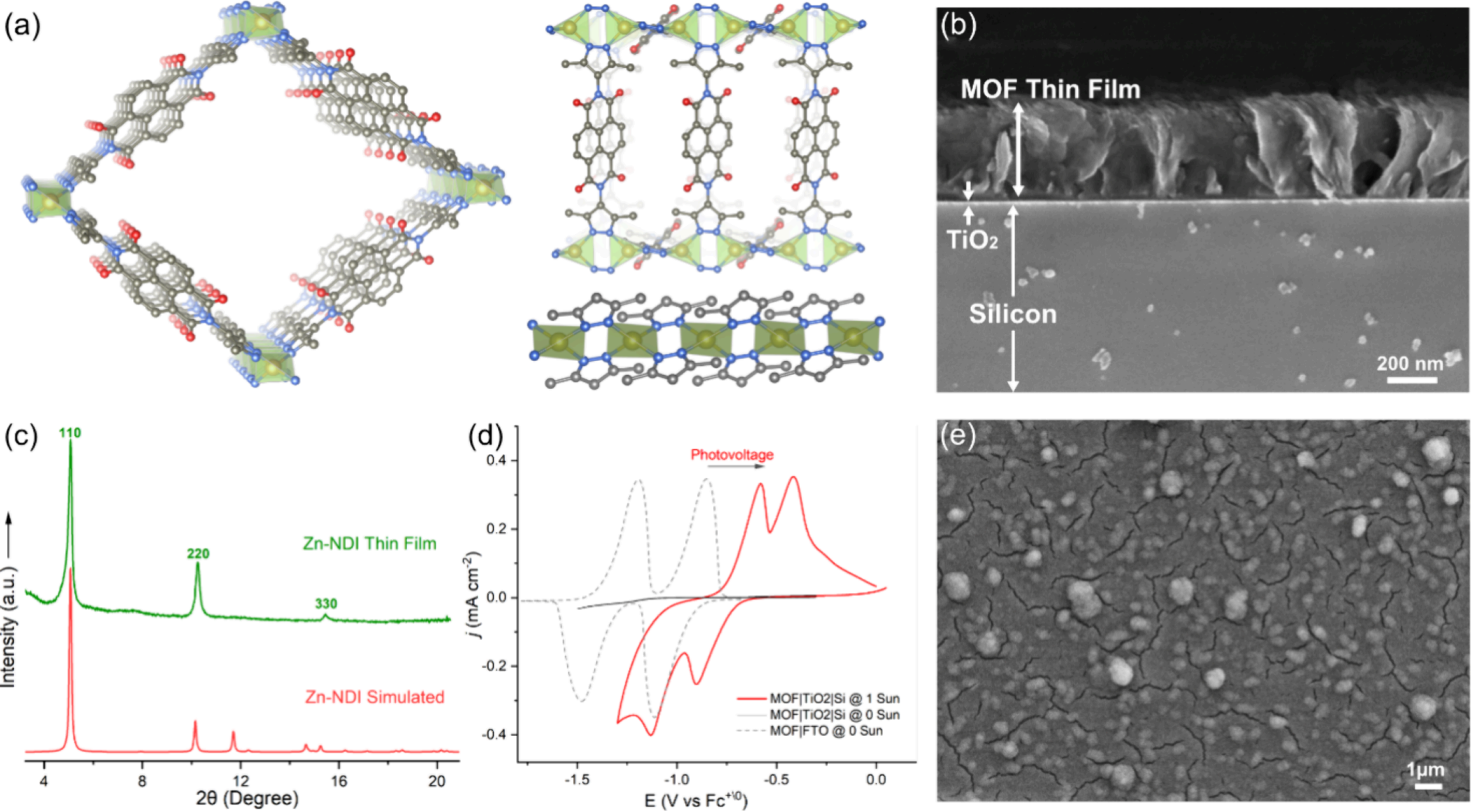
(a) Simulated crystal structure of the Zn-NDI MOF (constructed based on the reported structure in ref 24) viewing along the *c* direction (left), and neighboring NDI linkers and the pyrazolate-bridged Zn^2+^ chain (right). Hydrogen atoms are omitted for clarity; C, N, O, and Zn atoms are presented with gray, blue, red, and yellow spheres, respectively. (b) SEM cross-section image of an employed Zn-NDI|TiO_2_|Si photocathode. (c) Thin-film X-ray diffraction (XRD) of the Zn-NDI|TiO_2_|Si (green) with preferred orientation, where simulated powder XRD (red) is included for comparison. (d) CV of Zn-NDI on FTO (black dashed line), a Zn-NDI|TiO_2_|Si working electrode @ 0 sun (black) and @ 1 Sun (AM 1.5G, red) at a scan rate of 25 mV s^–1^ with 0.5 M KClO_4_ in DMF as the supporting electrolyte. Under illumination, the half -wave potentials (*E*_1/2_) of both NDI/NDI^•–^ and NDI^•–^/NDI^2–^ redox pairs are shifted anodically. (e) Top view SEM image of Zn-NDI MOF film.

The Zn-NDI|TiO_2_|SC photocathode shows an efficient interfacial charge separation and an enhanced photovoltage, two important parameters for PEC technologies.^19^ The rate of electron hopping through the MOF layer can be quantified by the apparent electron diffusion coefficient (*D*_e_^app^), which is in the order of 10^–9^ to 10^–11^ cm^2^ s^–1^.^20–23^

## Results and Discussion

Thin films of Zn-NDI were directly grown onto TiO_2_-coated p-type SCs (gallium phosphide and silicon) under solvothermal conditions, following a previously reported procedure (see Supporting Information for details about SC cleaning, TiO_2_ coating, and MOF thin film synthesis).^25,26^ Briefly, the TiO_2_-coated SCs were exposed to a solution of Zn­(NO_3_)_2_ and the NDI linker in DMF at 135 °C for 4.5 h. The resulting MOF forms as a compact film with a thickness of 350–400 nm, as determined by cross-sectional scanning electron microscopy (SEM; Figures 1b, S2, and S3). Top-view and cross-sectional SEM combined with energy dispersive X-ray spectroscopy (EDX) elemental mapping of the photocathodes further confirmed the uniform and homogeneous growth of MOF thin-film on the TiO_2_-coated Si and GaP surfaces (Figures 1e, S4, S5, S6, and S7). The incorporation of NDI linker into the framework was probed by attenuated total reflection Fourier transform-infrared spectroscopy (ATR-FTIR), which showed characteristic bands of Zn-NDI|TiO_2_|SC similar to those of the free linker. The main difference between the spectra is an N–H stretching vibration that is visible in the FTIR spectrum of the free linker but absent in the Zn-NDI|TiO_2_|SC due to deprotonation of the pyrazole anchor groups and coordination to Zn^2+^ (Figures S8 and S9). Functionalization of the SC surface was also examined by X-ray photoelectron spectroscopy (XPS) which displayed all the major peaks corresponding to the Zn-NDI MOF, verifying the presence of uniformly grown MOF thin-films without significant pinholes (Figures S10 and S11). The crystallinity and structural features of the thin film are confirmed through thin-film X-ray diffraction (XRD). Compared to the simulated powder XRD pattern, the thin film sample exhibits a high degree of preferred orientation on the Si surface with distinct (110), (220), and (330) reflection (Figure 1c).^26^ Photoelectrochemical properties of the Zn-NDI|TiO_2_|Si photoelectrodes were tested in a three-electrode configuration, using a solar simulator as the light source and 0.5 M KClO_4_ in DMF as electrolyte (more details in Supporting Information). At 1 sun illumination, two redox events with midpoint potentials at −0.65 and −0.85 V vs Fc^+/0^ are clearly observed (Figure 1d, red curve), corresponding to two consecutive, one-electron redox processes, NDI/NDI^•–^ and NDI^•–^/NDI^2–^, respectively. The first and second redox potentials are shifted anodically by 320 mV and 490 mV, respectively, compared to the two formal potentials (−0.97 and −1.34 V vs Fc^+/0^) of Zn-NDI@FTO (Figure 1d, dashed curve; see Supporting Information for electrode preparation and electrochemical characterizations).^15^ In the dark, cyclic voltammograms (CVs) of the Zn-NDI|TiO_2_|Si showed little to no current response in the same potential range (Figure 1d, black curve), indicating that photogenerated electrons are required for MOF reduction. In essence, the redox-active NDI linkers that the MOF is composed of act as acceptors for the photogenerated electrons.

In order to identify limiting phenomena in SC-surface layer systems such as the Zn-NDI |TiO_2_|Si photocathode, linear sweep voltammograms (LSVs) were recorded under varying light intensities. As indicated in Figure 2a, two qualitatively different LSVs of the Zn-NDI |TiO_2_|Si photoelectrode can be observed depending on the illumination intensity. At lower light exposure (below 5 mW cm^–2^), the LSVs are characterized by S-shaped waveforms with increasing plateau currents that correlate linearly with increasing light intensities (Figure 2a and Figure 2b, green region). When the illumination intensity exceeds 5 mW cm^–2^, peak-shaped waveforms that are characteristic of diffusional electrochemical responses take over. The electrochemical response is reminiscent of the CV of Zn-NDI|TiO_2_|Si under one-sun illumination (Figure 1d). In analogy, the peak-shaped features in the LSVs of Zn-NDI|TiO_2_|Si at illumination intensities beyond 5 mW cm^–2^ are thus assigned to the two consecutive one-electron reductions of the NDI linkers. Upon increasing light intensities, the peak positions keep shifting anodically (Figure 2a) while the peak currents remain basically constant (Figure 2b, pink region). The continuous anodic shift of the peak positions with increasing light intensities is in line with the movement of the onset potentials at an increased illumination intensity, which is related to the increase in photovoltage. Fundamentally, this phenomenon is the result of accelerated depletion of the neutral NDI species due to intensified photoelectron injection at the semiconductor-MOF interface.^27^ The illumination intensity at which the S-shaped waveform transitions into peak-shaped, diffusional waves in Zn-NDI|TiO_2_|Si is at ca. 5 mW cm^–2^.

**Figure 2:**
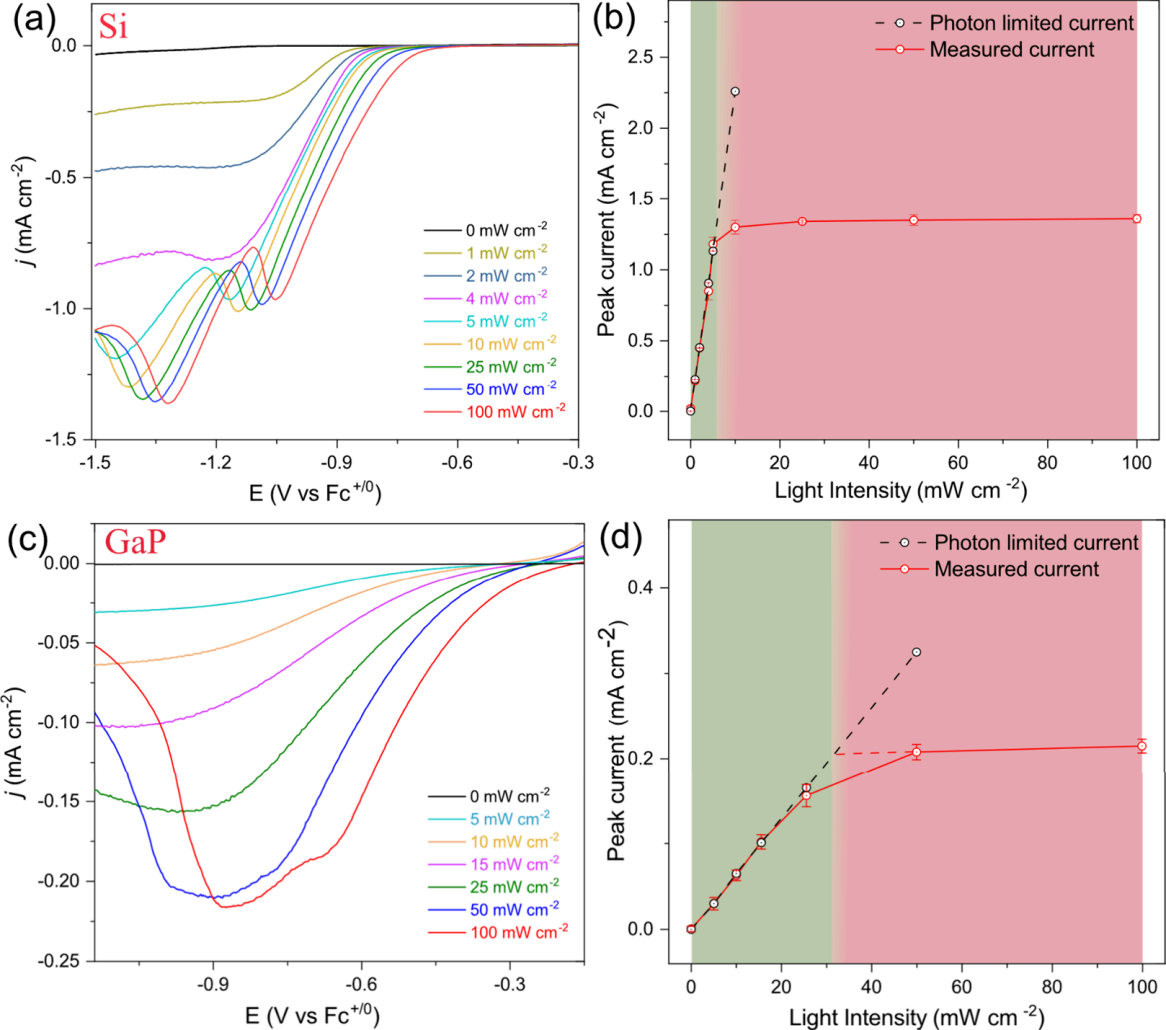
LSV of (a) Zn-NDI|TiO_2_|Si and (c) Zn-NDI|TiO_2_|GaP photocathode working electrodes (ν = 50 mV s^–1^, 0.5 M KClO_4_ in DMF) under varying illumination intensities, as indicated in the figure (1 sun = 100 mW cm^–2^). (b, d) Plots of the current densities as a function of light intensity. Green region represents regions of interfacial photoelectron-limited regimes, and pink region represents photoelectron diffusion-limited regimes. The dotted lines are extrapolations of the light intensity/current profiles in the two limiting regimes. Note that the CVs in (a) are measured at a higher scan rate compared to those in Figure 1d, resulting in anodically shifted cathodic peak potentials (*E*_p,c_) due to the dependence of Δ*E*_p_ on the scan rate.^26^ The *E*_1/2_ of the process is not dependent on the scan rate.

The different LSV shapes in the two regimes, as well as the change in light dependency, strongly point to a switch in the limiting phenomenon for photoelectron extraction by the Zn-NDI layer. The S-shaped waveforms with plateau currents under weak illuminations have a striking similarity with homogeneous catalytic systems under pure kinetic conditions and negligible substrate depletion.^28,29^ In analogy, such a behavior for the Zn-NDI|TiO_2_|Si photoelectrodes can be expected when the availability of photoelectrons at the photocathode–MOF interface remains constant during the cathodic sweep and when there is no limitation in terms of photoelectron extraction by the Zn-NDI acceptor layer. In other words, in the low-light regime, the concentration of NDI linkers at the photocathode–-MOF interface is sufficiently high to extract all available photoelectrons, and the MOF supports a sufficient electron flux to transport the photoelectrons away from the SC interface. Thus, the number of photoelectrons arriving at the interface is limiting the overall current density, also explaining the linear increase of current density with light intensity (Figure 2b, green region).

At higher light intensities, the situation changes. The supply of photoelectrons at the interface exceeds the maximum transport capacity of the MOF, and LSVs exhibit characteristic diffusion waveforms (Figure 2a, 5–100 mW cm^–2^). Diffusional waveforms are characteristic for redox-conducting MOFs, where electron transport proceeds by electron diffusion, i.e., electron hopping between immobilized redox active sites. The efficiency of this process can be quantified by an apparent electron diffusion coefficient, *D*_e_^app^, often in the range of 10^–10^–10^–11^ cm^2^ s^–1^. Overall, the observed dependence between LSV waveforms on different illumination intensities presented in Figure 2a can be categorized into two regimes: interfacial photoelectron limited regimes (S-shaped waveforms) and photoelectron diffusion limited regimes (characteristic diffusional waveforms).

To further explore the interplay between the two regimes for photoelectron extraction, the PEC characteristics of the p-GaP-based photocathode Zn-NDI|TiO_2_|GaP was investigated. p-GaP is an SC with a wider band gap compared to p-type Si, with a direct band gap up to 450 nm and an indirect band gap of 549 nm. The wider band gap results in a smaller part of the solar spectrum being absorbed, and the number of generated photoelectrons is about 1 order of magnitude lower compared to that in p-Si (Figure S12).^30^ For the Zn-NDI|TiO_2_|GaP systems, the lower availability of photoelectrons should extend the interfacial photoelectron limited regime to higher light intensities. This is indeed the case, and the current densities of the Zn-NDI|TiO_2_|GaP photocathode increase linearly with light intensity up to a threshold of ca. 31 mW cm^–2^. Beyond this light intensity, the surface layer-based electron diffusion-controlled regime becomes dominant, and consequently, the current densities cease to increase with increasing light intensities (Figure 2c,d). This indicates that by replacing Si with GaP as the light absorber, it is possible to modulate the transition point of the two different regimes, highlighting the fact that the photoelectron diffusion process within the surface MOF thin film has to be considered when evaluating the overall PEC performance of the photoelectrodes. The switch in limiting regimes is not only a phenomenon that is intrinsic to the Zn-NDI MOF but has also been observed in a porous interpenetrated Zr-organic framework (PIZOF) with integral NDI-based linkers (Figure S17).

To delve deeper into the importance of photoelectron transport in the MOF-based PEC constructs, the rate of charge transport was modulated by varying the *D*_e_^app^, and its effect on the two limiting regimes was analyzed. Increased *D*_e_^app^ should decrease the photoelectron transport limitation, and the transition light intensity between the two limiting regimes should move to a higher value. MOFs are ideally suited for this purpose, as their transport properties, and thus *D*_e_^app^, are sensitive to solvent and electrolyte, which in turn alter ion pairing between reduced linkers and electrolyte cations, and solvation in general.^31^ We have previously demonstrated that a change of solvent can boost the *D*_e_^app^(KCl_(aq)_) up to 1 order of magnitude.^32^ In the present case, independent measurements of *D*_e_^app^ under aqueous conditions for the MOF-modified semiconductors resulted in a ca. 4-fold increase compared to that measured in DMF (with 0.5 M KClO_4_ as supporting electrolyte) (Figures S13 and S14). A complication of the Zn-NDI MOF is that the two well-separated reductions that are observed in DMF collapse into one wave in an aqueous electrolyte, which however does not affect the subsequent discussion. As expected, the measured peak current densities for both Zn-NDI|TiO_2_|SC photoelectrodes saturate at higher light intensities (Figure 3a,d) compared to those of the analogous experiments under nonaqueous conditions (Figure 2). Specifically, for the GaP-based photocathode, the photon-limited regime now extends until 41 mW cm^–2^, an increase of around 33% compared to the value measured in DMF (Figure 3e,f). The same picture emerges for the Zn-NDI|TiO_2_|Si substrate and a proportional increase of the photon-limited regimes toward higher light intensities by around 34% is observed (Figure 3b,c). The shift of saturation currents to higher illumination intensities suggests that it is possible to extend the interfacial photoelectron-limited regimes by increasing the *D*_e_^app^ of the MOF. This means that designing redox-conducting MOFs with a higher intrinsic *D*_e_^app^ can further extend the interfacial photoelectron-limiting regimes, thereby suppressing diffusional limitations of the photoelectrons. Encouragingly, there are several reported strategies to improve the *D*_e_^app^ of redox-active MOFs.^33–35^ Additionally, a catalytic MOF could also promote higher current densities by consuming the photogenerated charges into a final product, even with a *D*_e_^app^ value comparable to the ones herein.^23,36,37^ Meanwhile, there are also scenarios in which maximum current density is not the primary goal, for example, in a multielectron, multiproton reactions in which the product selectivity is valued over the catalytic rate.^38,39^ In such systems, the controlled availability of electrons may give kinetic intermediates of a catalytic cycle sufficient time to convert to the thermodynamic product, thereby altering product speciation.

**Figure 3:**
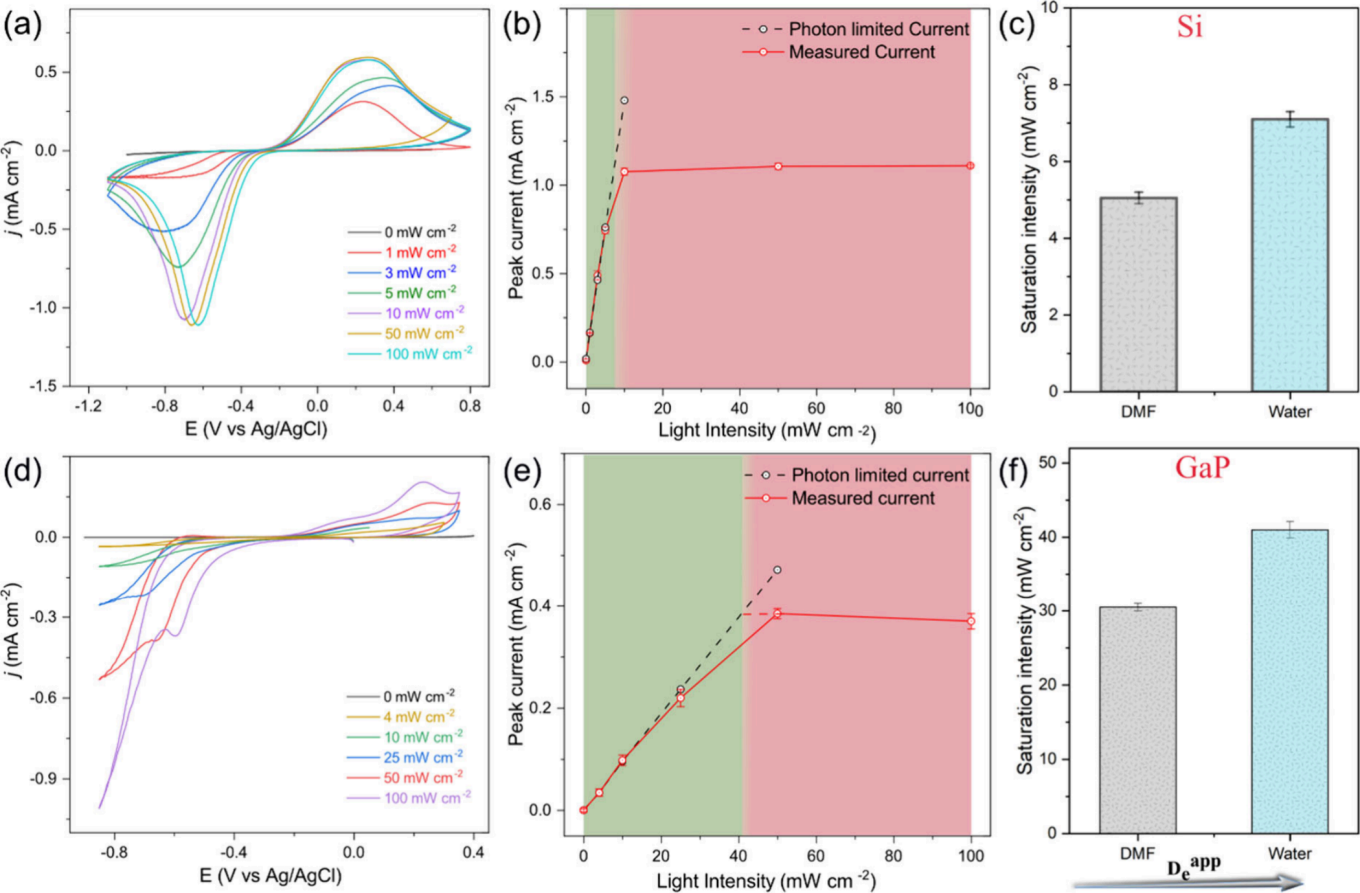
CVs of (a) Zn-NDI|TiO_2_|Si and (d) Zn-NDI|TiO_2_|GaP photocathode working electrodes (ν = 50 mV s^–1^, 0.5 M KCl in H_2_O) under varying illumination intensities, as indicated in the figure (1 sun = 100 mW cm^–2^). (b, e) Plots of the corresponding current densities as a function of light intensities. The green region represents the interfacial photoelectron-limited and the pink region the photoelectron diffusion-limited regime. The dotted lines are extrapolations of the light intensity/current profiles of the two limiting regimes. (c, f) Illustration of how the light intensity at the transition between the two limiting regimes correlates with the electron transport properties of the Zn-NDI layer, as characterized by the apparent electron diffusion coefficient, *D*_e_^app^. Water as solvent sustains higher *D*_e_^app^ values, and consequently, the photocathodes are under interfacial photoelectron-limitation up to higher transition light intensities.

## Conclusion

In summary, using a well-defined redox-active Zn-NDI MOF-integrated photocathode as a model system, factors that are limiting the overall photoelectrochemical performance are analyzed. On top of the interfacial photoelectron limited region, an additional photoelectron diffusion limited region was identified at higher illumination intensities. The latter is fundamentally related to the cation-coupled photoelectron hopping transport nature in the MOF thin film and can be modulated by changing the underlying semiconductor (from p-Si to p-GaP) or changing the apparent electron diffusion coefficient, *D*_e_^app^, by virtue of the solvent and supporting electrolyte. This transition from interfacial photoelectron limitations to transport limitations in the surface layer adds to the conventional views on the kinetic limiting factors of photoelectrodes. Photocarrier diffusion in the surface modification layer of a photoelectrode has to be considered, especially for thick overlayers such as cocatalysts, redox polymers, and porous materials.

## Supplementary Material



## Notes

Detailed experimental procedures, synthetic details, additional characterization of the photoelectrodes, Cottrell analysis, photoelectrode stability, and electrochemically active surface area (PDF)
