# Supplementary material for: Switching between Limiting Charge Extraction Regimes in an Illuminated Semiconductor–Metal–Organic Framework Junction
Source: J Am Chem Soc. 2025 Jun 10;147(25):21996–2002. doi: 10.1021/jacs.5c05700 (PMC12203599; doi:10.1021/jacs.5c05700)
Supplement: Supplementary file 1 [file ja5c05700_si_001.pdf]

Supporting information

for

Switching between Limiting Charge Extraction Regimes in an Illuminated Semiconductor - Metal-organic Framework Junction

Amol Kumar[1], Jingguo Li,[1,2,]* Anna M. Beiler,[1] and Sascha Ott[1,]*

[1] Department of Chemistry – Ångström Laboratory, Uppsala University, Box 523, 75237 Uppsala, Sweden

[2] Department of Environmental Science and Engineering, University of Science and Technology of China, Hefei 230026, China

* Corresponding authors: lijg@ustc.edu.cn; Sascha.Ott@kemi.uu.se

Contents

General information: Materials and Instrumentation 3
Materials 3
Instrumentation 3
Experimental Section 5
Preparation of working electrode: 5
Simulated Zn-NDI structure: 6
Characterization of Photo-electrode 7
SEM: 7
SEM-EDX: 8
ATR-FTIR: 12
XPS analysis: 14
Photo electrochemical measurements: 16
Solar Irradiance Plot: 17
Cyclic Voltammetry of MOF Thin Film on FTO 18
Cottrell Analysis 19
$Deapp$ determination to assess charge diffusion-migration in MOF films: 19
Photoelectrode stability 23
Electrochemically active surface area (EASA): 24
Switching between limiting regimes in a different PIZOF MOF thin film on GaP 24
Reference 25

General information: Materials and Instrumentation

Materials

All solvents and commercially supplied chemicals were reagent grade and used as received without further purification, unless stated otherwise. Zn $(NO_3)_2{\cdot}6H_2O$ (Sigma-Aldrich), naphthalene-1,4,5,8-tetracarboxylic dianhydride (≥97.0%) from TCI, 4-amino-3,5-dimethylpyrazole (≥95.0%) were purchased from Fluorochem and N, N-dimethylformamide (DMF) (99.9%) were purchased from VWR. Potassium hexafluorophosphate ($KClO_4$, for electrochemical analysis, ≥99.0%) and fluorine-doped tin oxide (FTO) substrates (7 Ω/sq) were purchased from Sigma-Aldrich. Both p-type (111) oriented Silicon (Si) wafers (thickness ~ 280 ± 25 µm) and p-type (100) oriented Gallium phosphide (GaP) wafer (thickness ~ 415 ± 25 µm) used for working electrode were purchased from ITME. Gallium-indium (Ga-In) eutectic (99.99%) was purchased from Sigma-Aldrich.

Instrumentation

Atomic layer deposition (ALD): Atomic layer deposition of TiO_2 was performed on Si and GaP wafers using a Picosun R-200 ALD system at 200 °C. The film was grown with alternating cycles of the $TiCl_4$ and H_2O precursors, with $TiCl_4$ as the titanium source and water vapor as the oxygen source.

Powder X-Ray Diffraction (PXRD): Thin film XRD measurements were performed on a Bruker D8 Advance diffractometer (D8Powder) using a monochromatic Cu Kα radiation (λ = 1.5406 Å) source operating at 40 kV and 40 mA. PXRD data was collected between 3 and 24^0 (2θ) range at a step size of 0.017^o in Bragg-Brentano geometry with a Lynxeye XE-T (solid state strip detector) position sensitive detector.

Scanning Electron Microscopy (SEM): Scanning electron microscopy (SEM) images were obtained using a Zeiss 1550 Schottky field emission scanning electron microscope equipped with an in-lens detector operated at 1 – 30 kV acceleration voltage. MOF thin film were anchored to conductive carbon tape on a sample holder disk and connected to the underlying sample holder with conductive Aluminium tape from the top side of the samples. Surface area and film thickness of each individual film was determined using ImageJ (NIH) software.

SEM-Energy Dispersive X-ray Spectroscopy (EDX): EDX data were collected with an 80 mm^2 Silicon Drift Detector using AZtec (INCA energy) software at an acceleration voltage of 6-8 kV and a working distance of 7-9 mm.

Attenuated Total Reflectance-Fourier Transform Infrared spectroscopy (ATR-FTIR): ATR-FTIR data were collected on a Bruker 70v FTIR spectrometer from 4000 to 700 cm^{-1} at room temperature.

X-ray photoelectron spectroscopy (XPS): XPS was performed on a PHI Quantera ll scanning XPS microprobe using a monochromatic Al Kα source (hν = 1486.6 eV). For a homogeneously overgrown sample it was operated at 25 W and a beam diameter of 200 µM (survey). The presented survey scans were recorded in a binding energy range of 1200–0 eV with a pass energy of 224 eV, 50 ms per step, and with 10 cycles. Data were analyzed using Multipak, and all spectra were calibrated by setting adventitious carbon to 284.6 eV.

Experimental Section

Preparation of working electrode:

Boron-doped p-type (111) oriented Si with resistivity 0.3- 0.5 Ω cm and Zn-doped p-type (100) oriented GaP wafers with a resistivity 0.20- 0.21 Ω cm, a dopant concentration of 3.5 -3.8 × 10^{17} cm^{-3} was used as the model substrate with an active area of 0.28 cm^2. Before atomic layer deposition (ALD), Si and GaP wafers were cleaned and etched to remove contaminants and native oxide layer (details discussed below). After that, a layer of TiO_2 was deposited through ALD at 200 °C on the Si/GaP wafers with $TiCl_4$ as the titanium source and water vapor as the oxygen source in pulse mode under an argon flow. The thickness of the TiO_2 film can be regulated by controlling the number of ALD cycle. Using ellipsometry, it was established that 100 cycles of ALD produce a 4 nm thick TiO_2 film, corresponding to a growth rate of ~0.4 Å per cycle at 200 °C. Based on this growth rate, applying 25 cycles in this study produced a TiO_2 layer of ca. 1 nm thick (Figure1)[1]. The synthesis of NDI linker and MOF thin film on TiO_2-coated p-type semiconductor (GaP and Si) follows a previously reported procedure.[2] Substrates after ALD were placed in a 20 ml scintillation vial with the coated side facing down. To this vial, DMF solutions of NDI linker (0.10 mmol), and Zn $(NO_3)_2{\cdot}6H_2O$ (0.11 mmol) were added. The vial was then placed in a gravity convection oven for 4.5 hours at 135°C. After solvothermal synthesis, the vials were allowed to cool to room temperature, and the MOF modified semiconductor wafers (Zn-NDI|TiO_2|SC) were washed with DMF to remove any loosely bound MOF.

Si wafer cleaning: p-type (111) oriented Si wafers were first sonicated sequentially in acetone, dichloromethane, and water for 5 minutes each to remove the organic and inorganic contaminations. The wafers were then etched in ammonium fluoride (40% solution) for 20 min to remove the native oxide layer followed by rinsing with deionized water (3-4 times) and dried under a stream of argon and stored in an argon environment. The wafers were then immediately brought into the ALD chamber for deposition of TiO_2 onto the surface.

GaP wafer cleaning: P-type (100) oriented GaP wafers were first cleaned by sonication for 5 min in acetone, followed by 5 min of sonication in isopropanol to remove organic and inorganic contaminations. To remove the native oxide layer, the wafers were etched in concentrated HCl for 1min and rinsed with deionized water (3-4 times) and dried under a stream

of argon. The wafers were then stored in an argon environment and immediately brought into ALD chamber for deposition of TiO_2 onto the surface.

Simulated Zn-NDI structure:

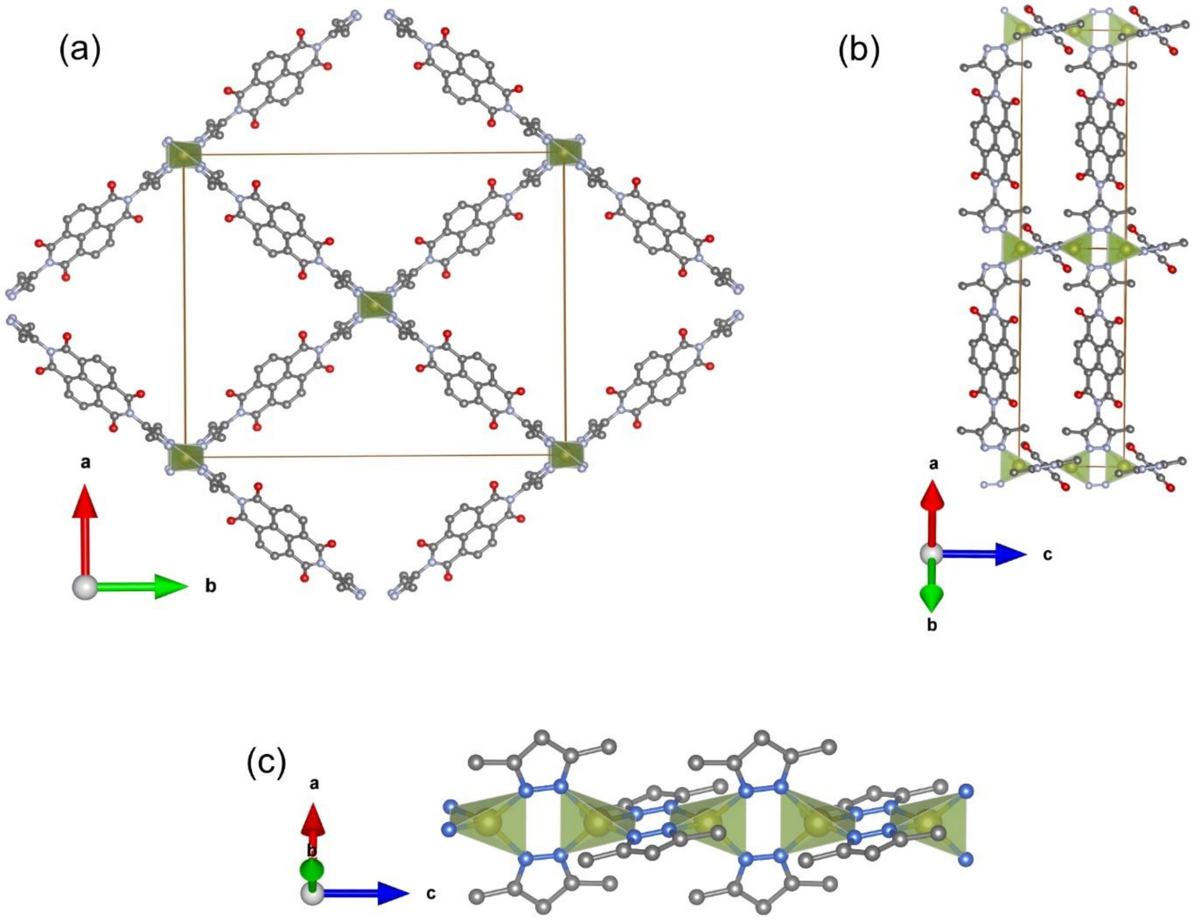

Figure S1. (a) Simulated crystal structure of Zn-NDI viewing along the c direction. (b) Rotated structure showing the neighboring NDI at a distance suitable for electron hopping. (c) The chain of Zn^{2+} cations with a focus on the coordination chemistry of the metal ions, showing the tetrahedral coordination of the Zn^{2+} metal ions (green polyhedra). Color code: Zn, yellow; N, blue; C, gray; O, red; H atom are omitted for clarity. The crystal structure was reconstructed based on the reported structure in ref [3].

Characterization of Photo-electrode

SEM:

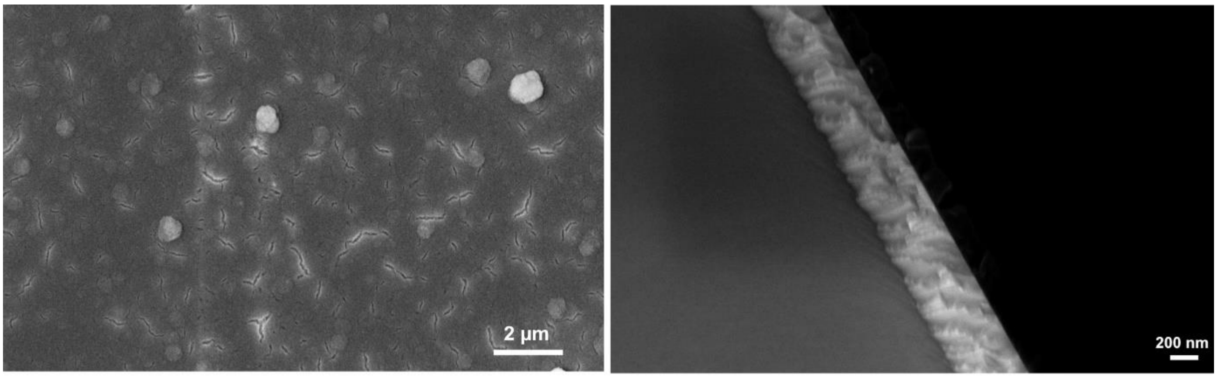

Figure S2. SEM top view image of the Zn-NDI|TiO_2|GaP photocathode (left). SEM cross-section (right).

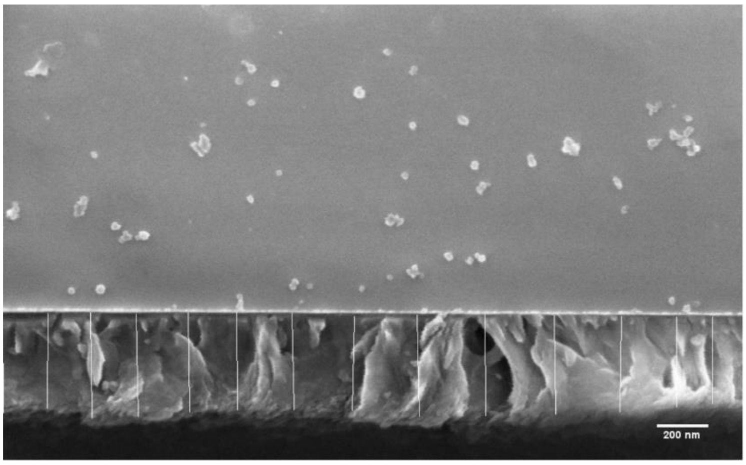

Measurement	Length (nm)
1	366.14
2	392.05
3	382.03
4	369.03
5	371.99
6	363.35
7	392.17
8	392.08
9	380.58
10	369.05
11	345.95
12	340.18

Figure S3. SEM cross-section image of the Zn-NDI|TiO_2|Si photocathode. The film thickness was determined at different points of the cross section, as indicated by the white bars (left). The measurements were done using the ImageJ program, and the results re listed in the column to the right.

SEM-EDX:

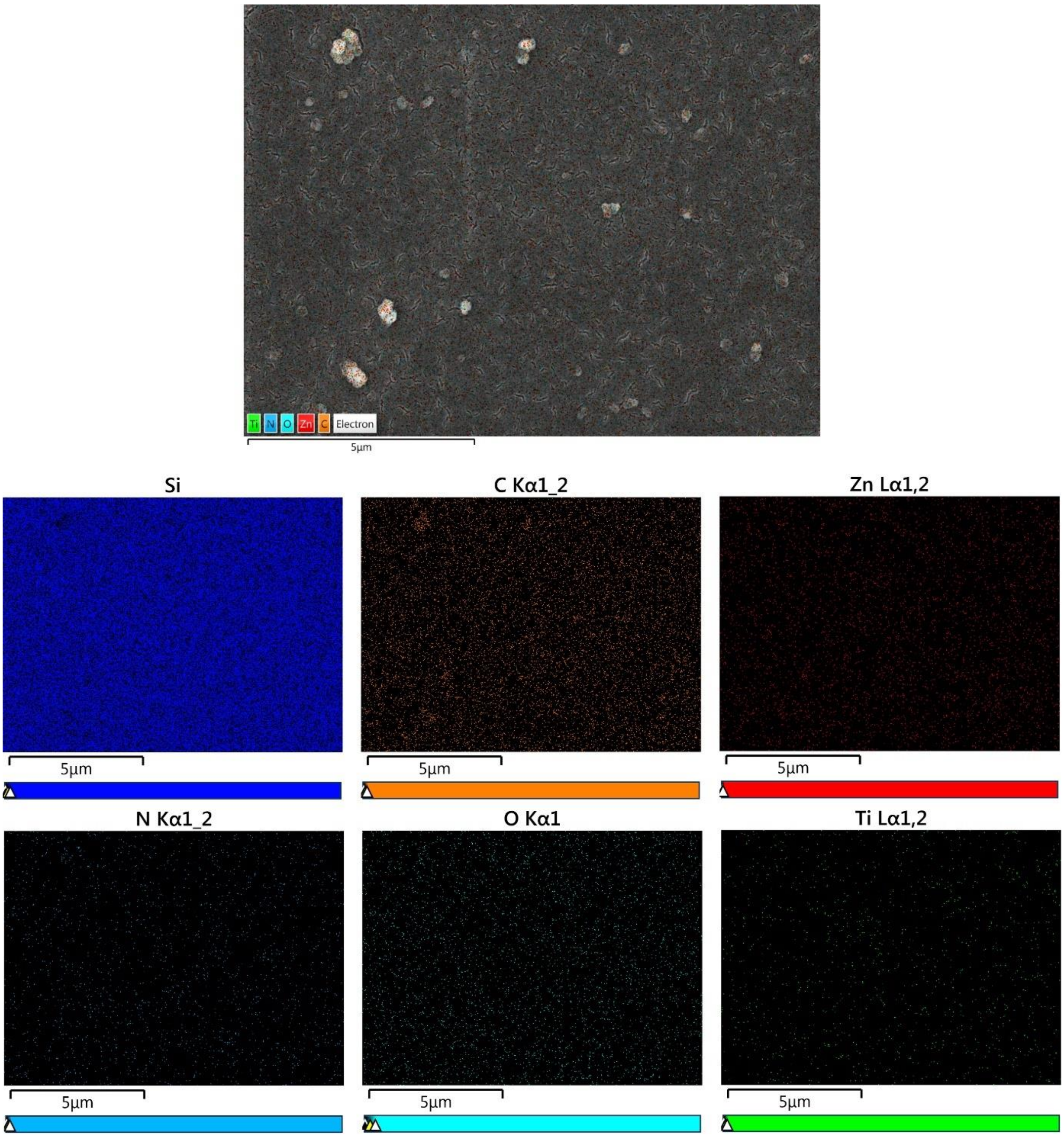

Figure S4. Representative top-surface SEM-EDX results for Zn-ND|TiO_2|Si. SEM and layered EDS image of Zn-ND|TiO_2|Si sample (top) and detailed map of scanned elements (bottom).

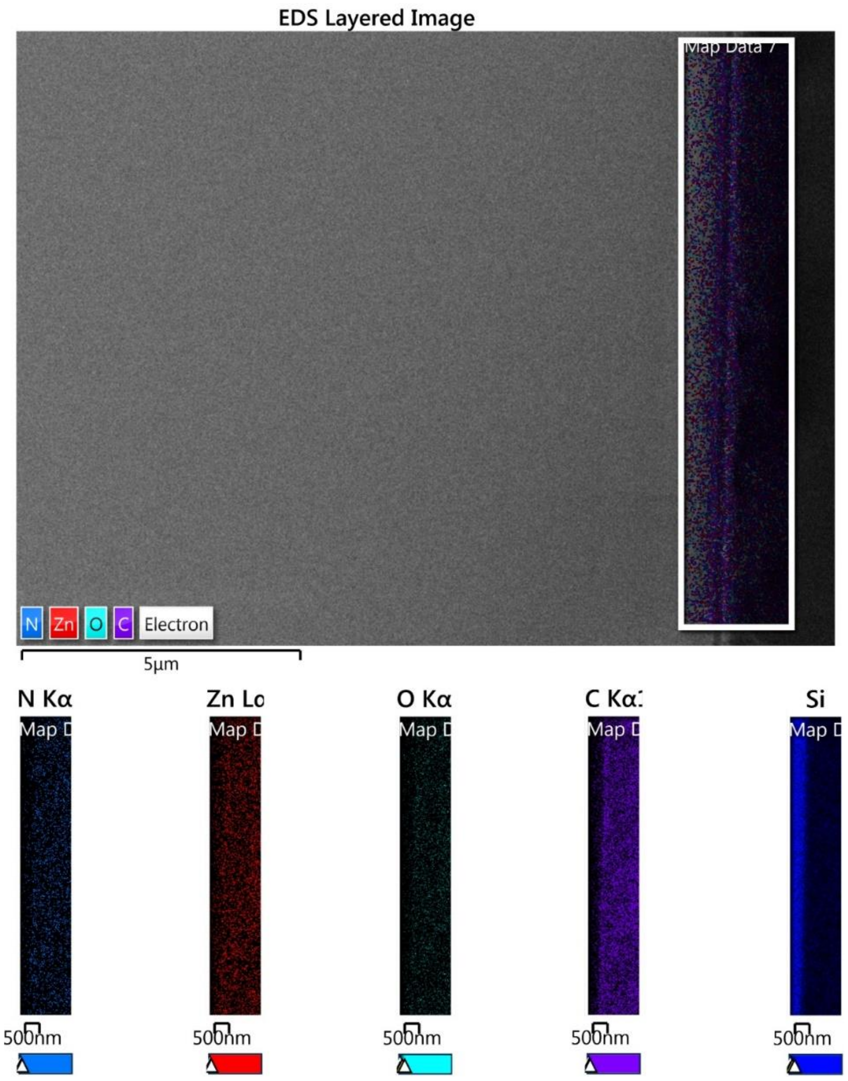

Figure S5. Representative cross-section SEM-EDX results for Zn-ND|TiO_2|Si. SEM and layered EDS image of Zn-ND|TiO_2|Si sample (in the White box) and detailed map of scanned elements (bottom).

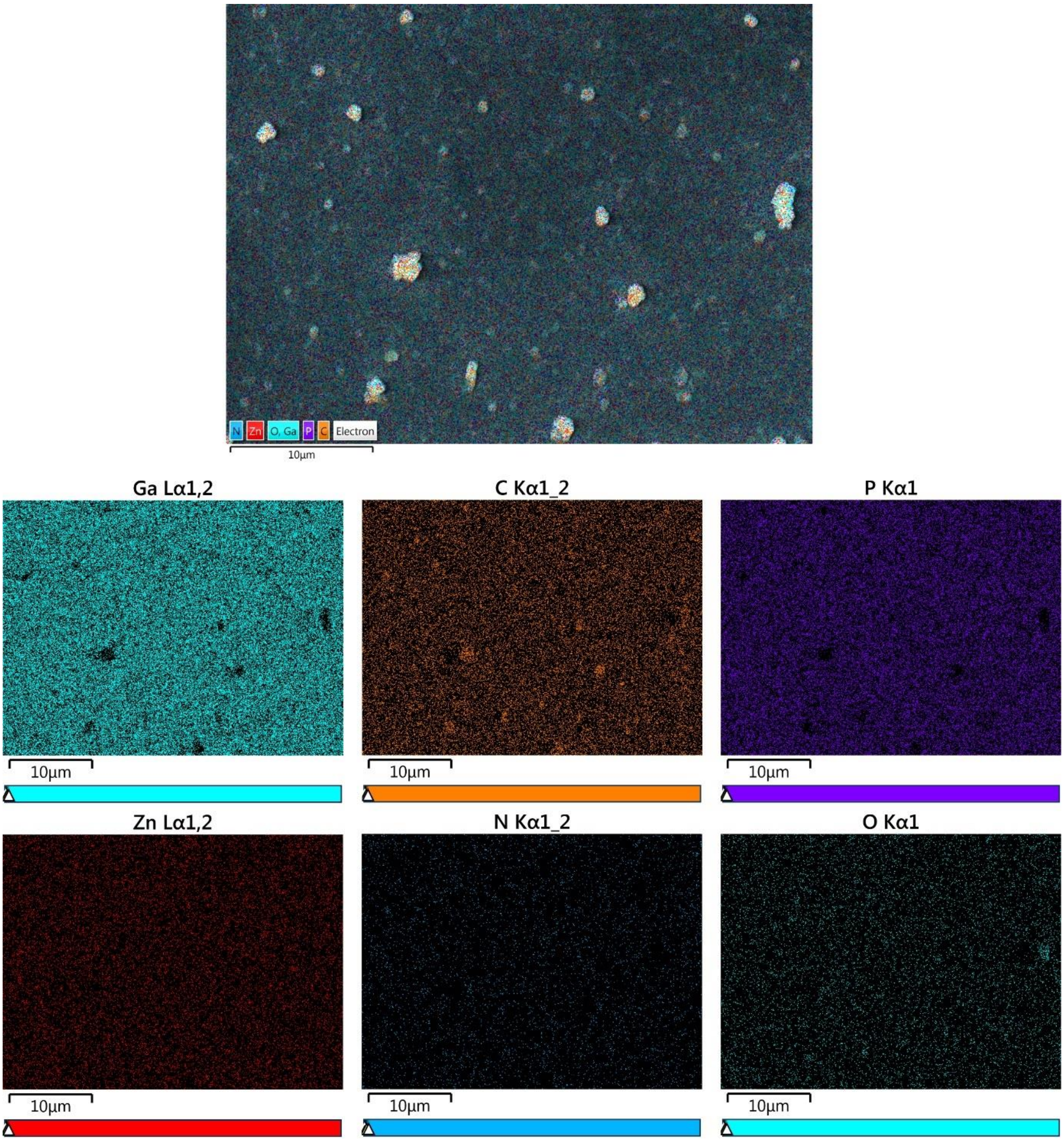

Figure S6. Representative Top-surface SEM-EDX results for Zn-ND|TiO_2|GaP. SEM and layered EDS image of Zn-ND|TiO_2|GaP sample (top) and detailed map of scanned element (bottom).

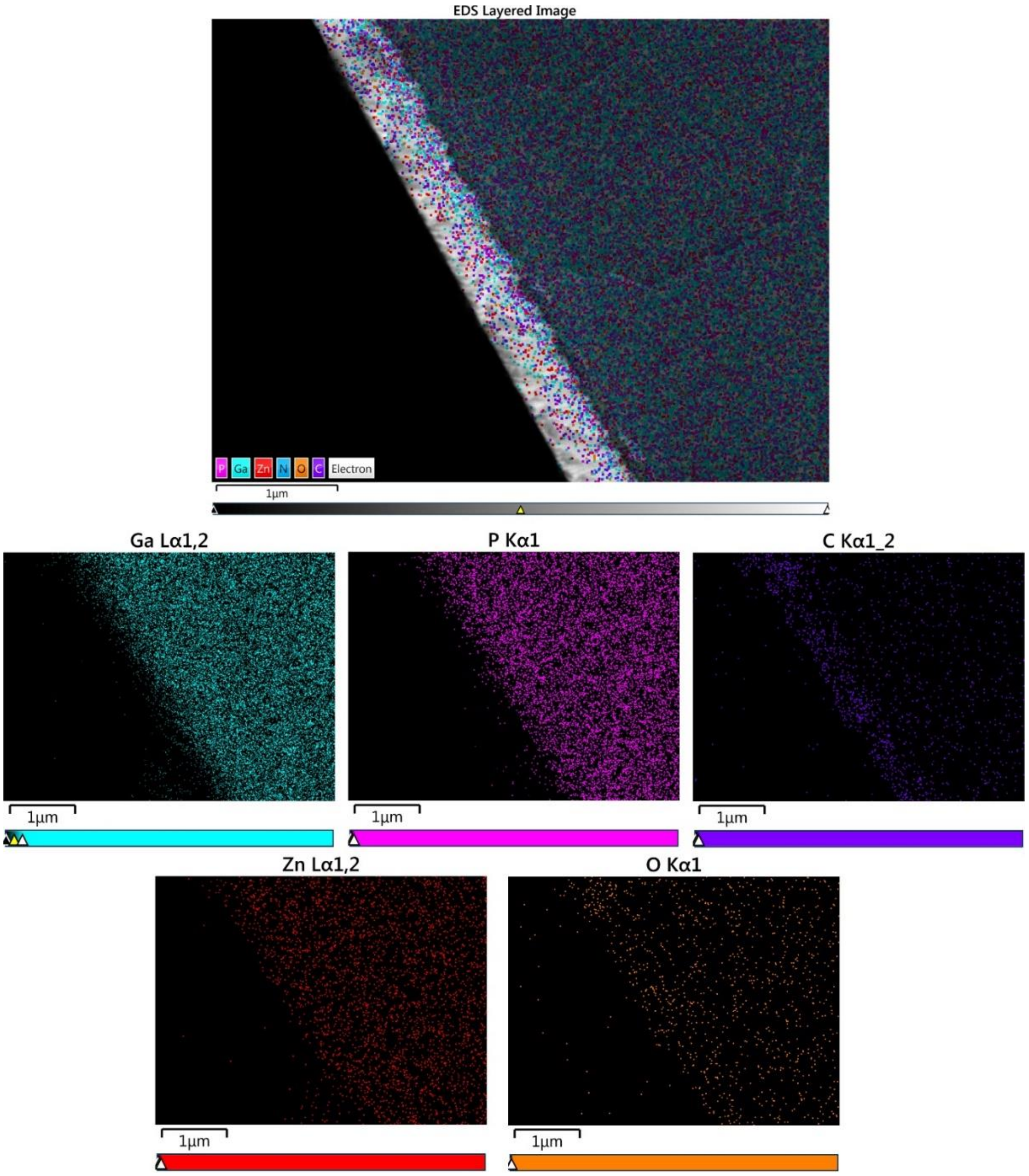

Figure S7. Representative Cross-section SEM-EDX results for Zn-ND|TiO_2|GaP. SEM and layered EDS image of Zn-ND|TiO_2|GaP sample (top) and detailed map of scanned element (bottom). The map of Ga and phosphorus in comparison with carbon shows the contrast between substrate (GaP) and Zn-NDI MOF thin film. As the p-type GaP is Zn-doped, Zn is present in the MOF as well as the substrate.

ATR-FTIR:

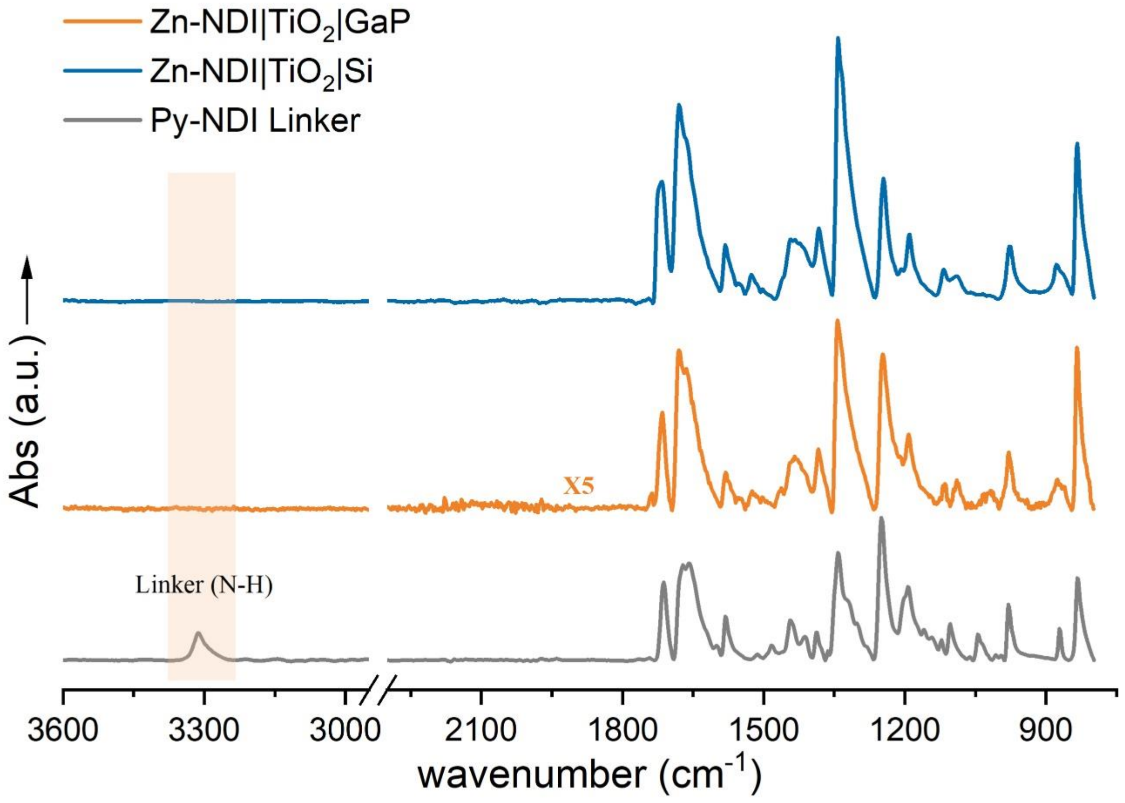

Figure S8. FTIR-ATR spectra of the free NDI linker and of the two photocathodes. The MOF thin films on the semiconductors exhibit vibrational bands similar to those of the free linker. Notably, the N-H peak of the free pyrazole linker around 3300 cm^{-1} is absent in the MOF as the pyrazole is deprotonated and binds to Zn^{2+} metal as pyrazolate.

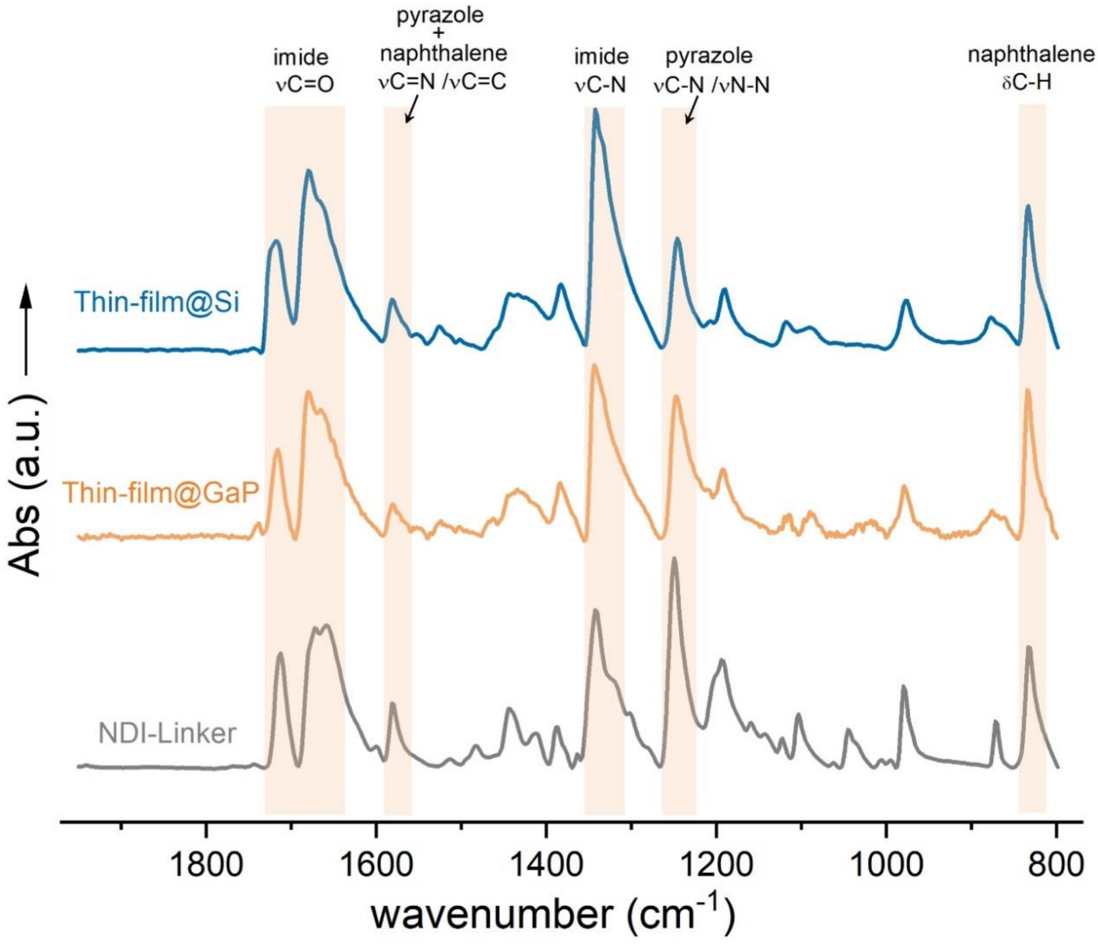

Figure S9. FTIR-ATR spectra of the free NDI linker and of the two photocathodes with assignments of some of the most prominent bands. The MOF thin films exhibit vibrational bands similar to those of the free linker, confirming the presence of structurally intact linker in the MOF. Visible are the asymmetric and symmetric C=O stretches of the imide group between 1750 and 1650 cm^{-1}, the peak at 1570 cm^{-1} is typical for conjugated π-systems; in the present case, the C=C stretches of the NDI core and/or C=N stretches of the pyrazolate ring. The peak in the range of 1390–1220 cm^{-1} confirms the presence of pyrazole and C–N bonds. The sharp peak at 1350 cm^{-1} is characteristic of C–N stretches in the imide unit of the NDI core. The peaks at 1250 cm^{-1} most likely reflect pyrazole-specific vibrations and are assigned for C-N and N-N stretching vibrations of the pyrazole ring, confirming the presence and integrity of the heterocycle. An interesting observation here is that relative intensity of this peak decreased after coordination to Zn in Zn-NDI|TiO_2|GaP and Zn-NDI|TiO_2|Si due to electron delocalization in the coordinated state suppressing N-N vibrations. The peak at 830 cm^{-1} supports the structural integrity and substitution pattern of the NDI aromatic unit and is assigned to aromatic C–H out-of-plane bending. The assignments are based on previous reports of the Zn^{2+}-pyrazolate motif and NDI-based materials.[4, 5]

XPS analysis:

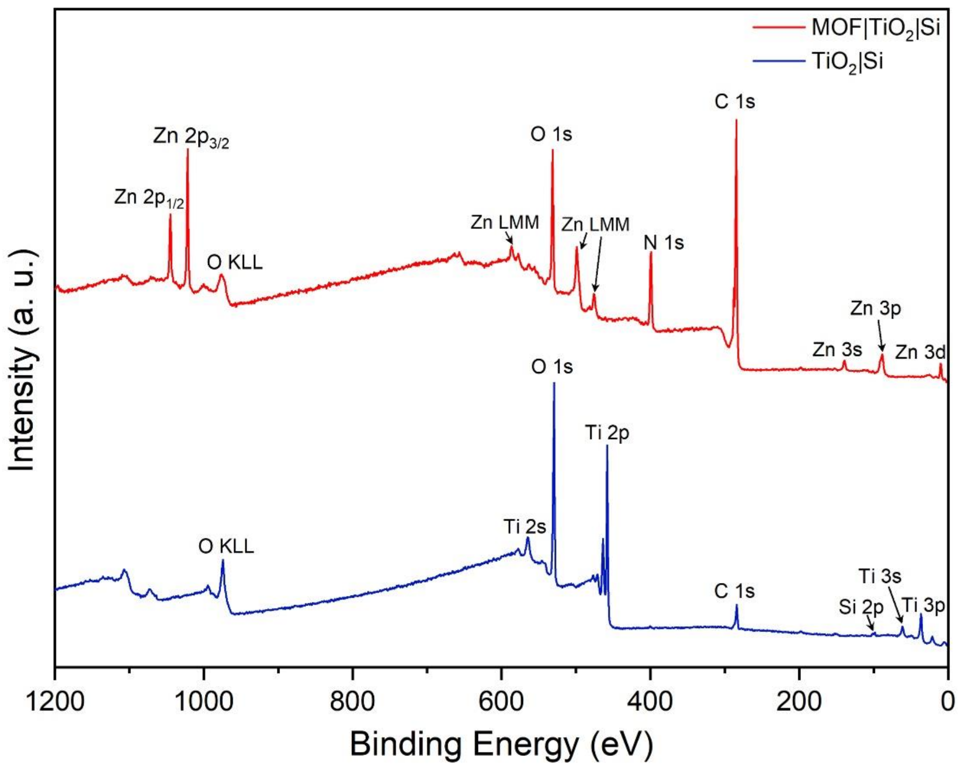

Figure S10. X-ray photoelectron spectroscopy (XPS) survey spectra of the Zn-NDI|TiO_2|Si photocathode (red), exhibiting all the major peaks of the Zn-NDI MOF (Zn, N, O and C) typically expected for homogeneously grown MOF thin-film. In contrast, the survey spectrum of the blank TiO_2 coated Si electrode, TiO_2|Si (blue), shows a strong signal for titanium and oxygen as well as a minor C 1s peak due to adventitious contaminations, and a very faint peak for silicon. The thickness of TiO_2 used for the blank here is 4-5 nm, matching the estimated penetration depth of XPS under the settings described in the instrumental section, explaining the predominance of Ti and O signals. In the case of MOF coated electrode, the MOF film thickness is on the order of hundreds of nanometers, far exceeding the XPS probing depth, resulting in the detection of MOF-derived elements only.

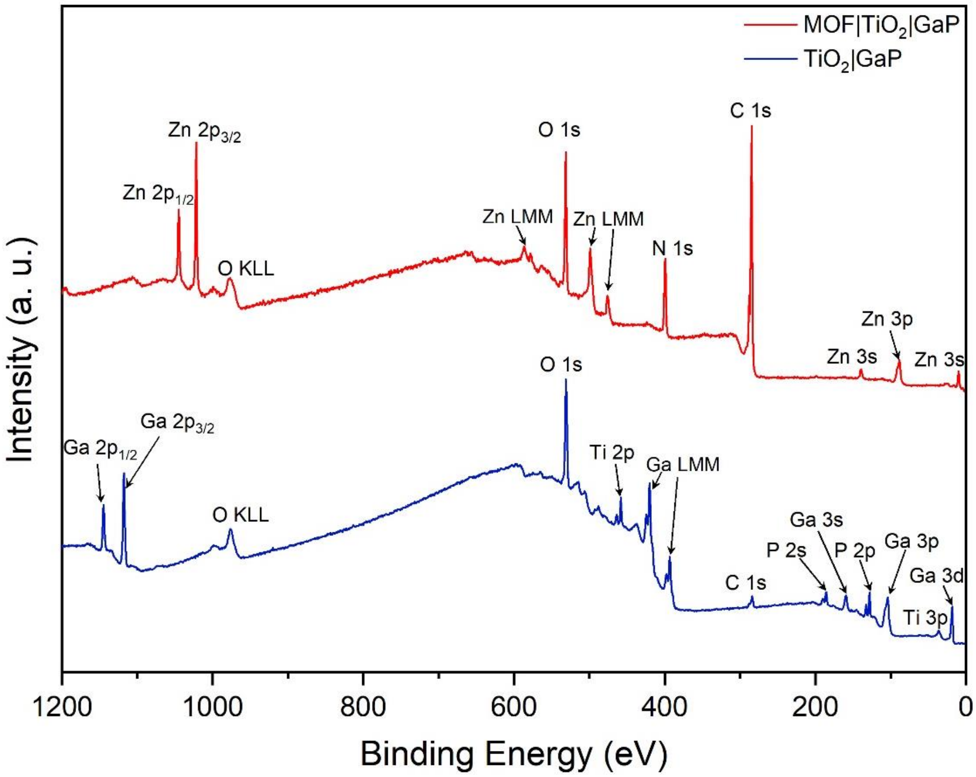

Figure S11. X-ray photoelectron spectroscopy (XPS) survey spectra of the Zn-NDI|TiO_2|GaP photocathode (red) with all the major peaks of Zn-NDI MOF (Zn, N, O and C) typically expected for homogeneously grown MOF thin film. In comparison, the survey spectrum of the blank TiO_2 coated GaP electrode TiO_2|GaP (blue) shows peaks for gallium, phosphorous, titanium and oxygen as well as a minor C 1s peak due to adventitious contaminations. The thickness of TiO_2 used for blank here is around 1 nm, thus less than the estimated penetration depth (4-5 nm) of XPS, explaining the signals from the TiO_2 as well as the underlaying GaP. In the MOF coated electrode, the MOF film thickness is on the order of hundreds of nanometers, far exceeding the XPS probing depth, resulting in the detection of only MOF-derived elements.

Photo electrochemical measurements:

Photo electrochemical analyses were performed in a three-electrode configuration using a commercially available pine cell (equipped with a quartz window). The functionalized SC wafer Zn-NDI|TiO_2|SC was used as the working electrode, a carbon rod as counter electrode, and non-aqueous $AgNO_3$ solution as reference electrode. The applied potential was calibrated against $Fc^{+/0}$ at the beginning and end of every experiment. A Ga−In eutectic film was painted on the back of the semiconductors to form an Ohmic contact with the stainless-steel back contact, the Ga−In contact was then connected to the external circuitry (Autolab PGSTAT204 potentiostat controlled with Nova 2.1.4 software) with the electrochemical cell. The illumination source was an Enlitech Solar Simulator with Xe light source and an AM 1.5 G spectral correction filter (Figure S14). The light intensity was calibrated using a NREL-traceable silicon reference cell before every experiment. Light intensity was further modulated (for intensity dependent measurement) using solar simulator and calibrated each time using silicon reference cell.

Solar Irradiance Plot:

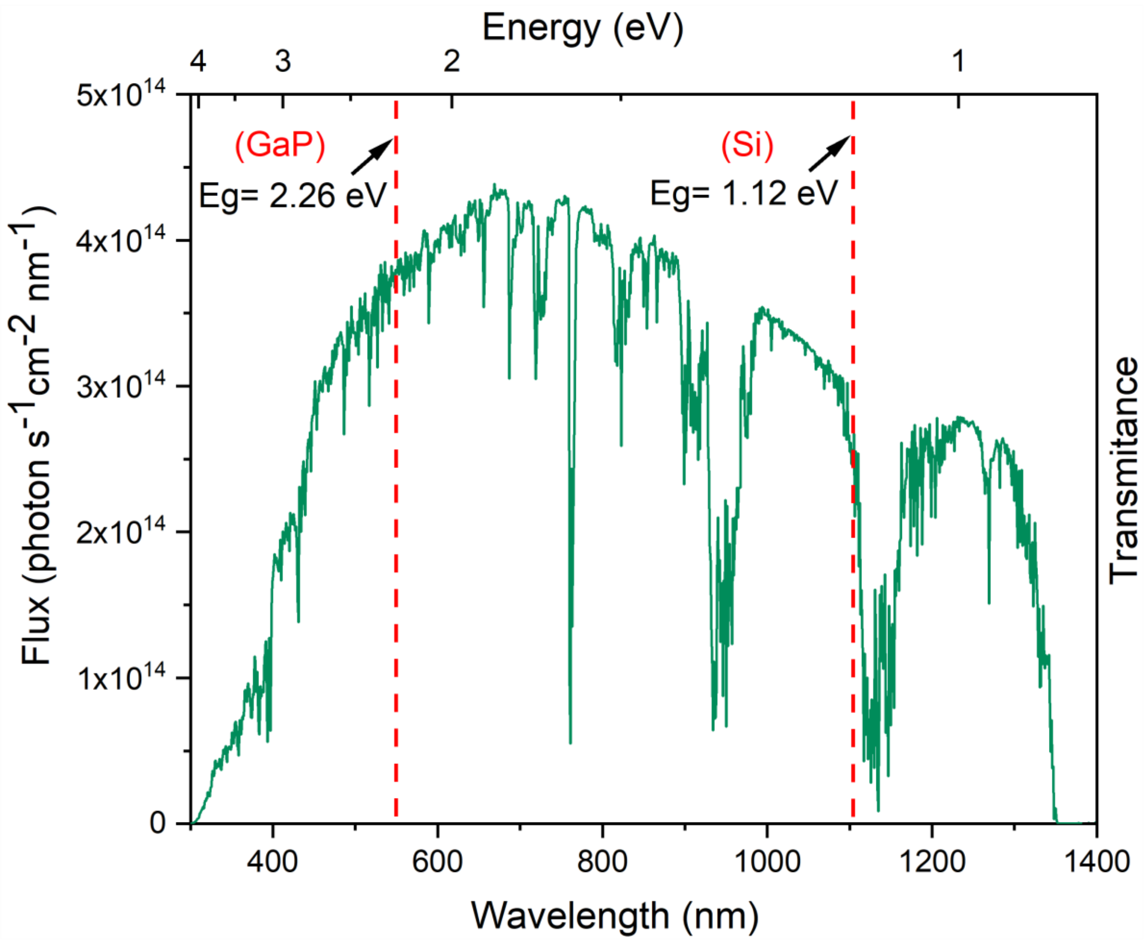

Figure S12. Solar irradiance plot[6]: 1Sun = 100 mW cm^{-2}, AM 1.5G. Absorbed photon for Si is 4.3 x 10^{17} Photon $s^{-1}cm^{-2}nm^{-1}$ and for GaP is 6 x 10^{16} Photon $s^{-1}cm^{-2}nm^{-1}$

Cyclic Voltammetry of MOF Thin Film on FTO

Electrochemical analyses were performed in a standard three-electrode set-up connected to an Autolab PGSTAT204 potentiostat controlled with Nova 2.1.4 software: the Zn-NDI@FTO was used as working electrode, a glassy carbon as the counter electrode, and a non-aqueous Ag/Ag^+ reference electrode (10 mM $AgPF_6$ in acetonitrile). Solutions of 0.5 M $KClO_4$ in dry DMF were used as the supporting electrolyte. MOF modified FTO was sonicated for 1 min to remove loosely bound partials prior to any experiments. The electrolytes were directly used as bought and the solvent was taken from the solvent purification system (SPS) without any further purification. Before the experiment, argon was bubbled for 15 min to the electrolyte solution. The head space of the electrochemical cell was continuously purged with argon during the experiments. The applied potentials were calibrated against the $Fc^{+/0}$ redox couple.

Cottrell Analysis

D_e^{app} determination to assess charge diffusion-migration in MOF films:

The apparent diffusion coefficient (D_e^{app}) in the different solvent aqueous and non-aqueous were determined by chronoamperometry. The data for analyzed Zn-NDI|TiO_2|SC films are shown in Figures S15-S16. After applying a sufficiently mild potential for 30s to begin with a neutral film, the potential was stepped to an appropriately negative potential to reduce the NDI linkers to the $NDI^{0/\bullet-}$ state and held for 100s. (Figures S15a, S16a) The current density (j) and recorded charge (Q) from this potential step were plotted vs. time to determine the total charge passed. A residual background current can be observed as the recorded charge does not decay to a slope of 0 after an extended time. This background current is subtracted by fitting the linear region of the charge (t = 65-90s after potential step for fits shown). (Figures S15b, S16b) The y-intercept of this linear fit line corresponds to the total charge passed to reduce all electrochemically addressable NDI linkers to the $NDI^{\bullet-}$ redox state in the film. The electroactive NDI concentration was calculated according to:

$$\Gamma_e = \frac{|Q|}{nFS_A} \quad \text{Eq. S1}$$

Where Γ_e is the electroactive linker concentration (in mol cm^{-2}), Q is the charge (in C), n is the number of electrons involved in the redox event, F is Faraday constant (in C mol^{-1}), and S_A is the surface area of the MOF-modified electrode (in cm^2). Here, it is assumed that n=1 (for DMF) as the applied potential is chosen to isolate the $NDI^{0/\bullet-}$ couple and n=2 (for H_2O) as both reduction events are merged. S_A is independently determined for each sample for accuracy. To determine D_e^{app} for electron-hopping diffusion in each system, the time-dependent current response (here identified as time-dependent current density j(t)) was fitted to the Cottrell equation:

$$j(t) = \frac{nF\Gamma e\sqrt{D_e^{app}}}{df\sqrt{\pi t}} \quad \text{Eq. S2}$$

Where d_f is the MOF film thickness (in cm^2) and D_e^{app} is the diffusion coefficient for charge diffusion in the film (in $cm^2 s^{-1}$). d_f for each film is measured from SEM images taken after electrochemical measurements. From a plot of j(t) vs. $t^{-1/2}$, it is evident that for points

corresponding to times shortly after the potential step, a linear correlation is observed (Figures S15d, S16d). These short time points correspond to a regime where the diffusion layer for electron-hopping charge diffusion is small and confined within the film, i.e. where diffusion is semi-infinite. The linear region (Cottrell region) to fit the data is determined through I$\sqrt{t}$ VS. log t plot.[7] In the absence of Ohmic and kinetic limitations, the Cottrell region appears in the I$\sqrt{t}$ vs. log t plot as a horizontal straight line. (Figures S15c, S16c) If, on the contrary, Ohmic and kinetic limitations affect the short-time current response, the Cottrell domain appears as a function with maximum or minimum, depending on the sign of the current.[8] The time domain obtained from I$\sqrt{t}$ vs. log t plot is then used for fitting linear region in plot of j(t) vs. $t^{-1/2}$. From fitting the linear region of this plot D_e^{app} can be extracted from the following relationship:

$$D_e^{app} = \left(\frac{\text{Slope} \ * \ \text{df}\sqrt{\pi}}{nF\Gamma e}\right)^2 \qquad \text{Eq. S3}$$

Experimentally determined values of D_e^{app} values for three films in both DMF and water were averaged, with a representative plot shown for each solvent below. (Figures S15-S16)

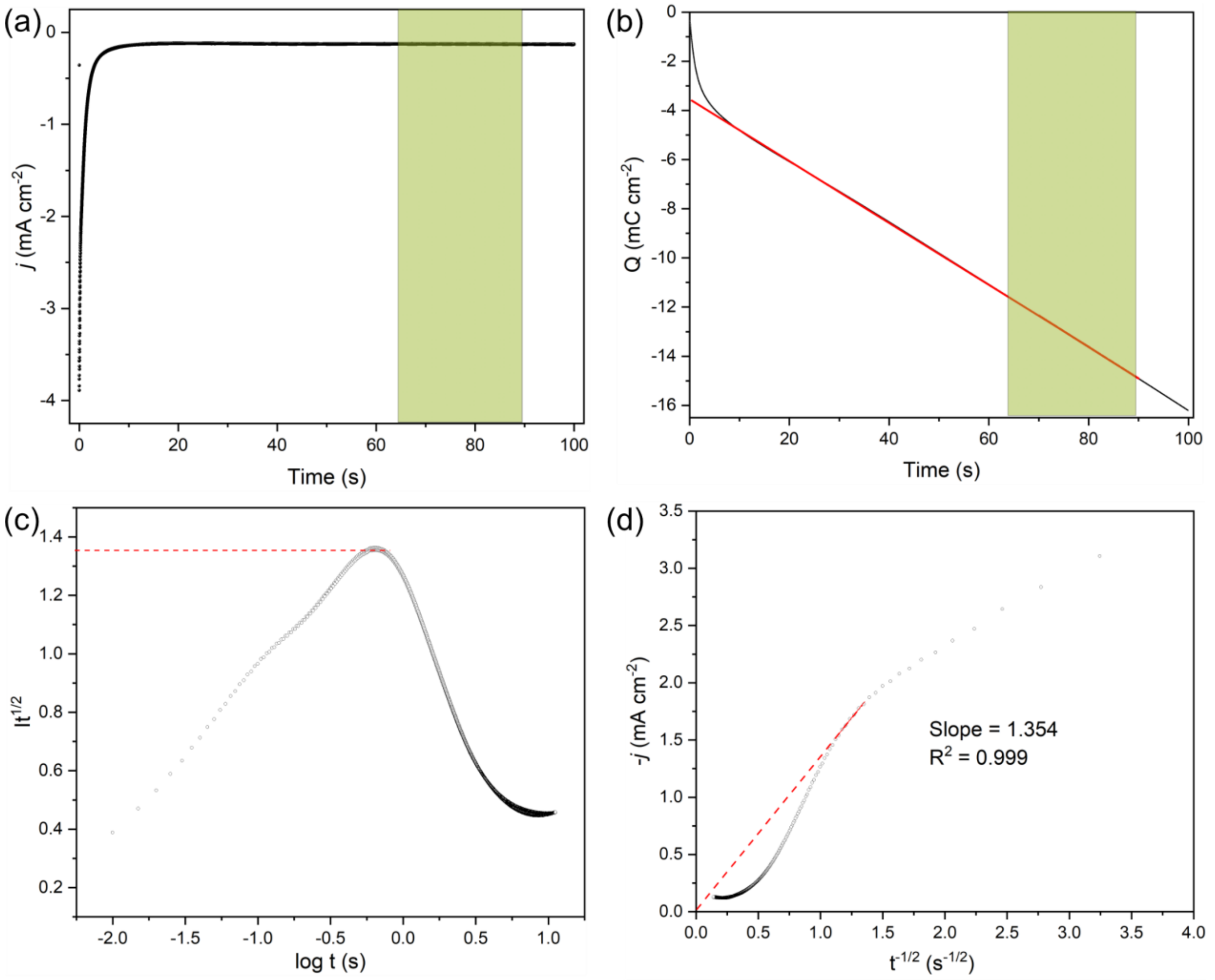

Figure S13. Electrochemical data for Zn-NDI|TiO_2|SC sample measured in 0.5 M KCl in H_2O. (a) Chronoamperometry (black circles) after stepping potential and (b) Chrono coulometry (black circles), the total charge passed after complete reduction of MOF film was determined after subtracting a residual background current (red solid line) Green boxes illustrate the window of time points chosen for linear fits for calculation of total surface concentration of redox active Unit. (According to Supplementary Eq.1) (c) Alternative representation of the Cottrell diffusion in the form of time dependence of the function $I(t)t^{1/2}$ calculated for the same data as in (figure S2, a), Dashed red horizontal lines define the maximum values of $I(t)t^{1/2}$ (*i.e.*, the Cottrell parameter) (d) Cottrell plot for Zn-NDI|TiO_2|SC measured in 0.5 M KCl in H_2O with a time step of 0.005 sec, After the potential step, the linear fit (red dashed line) used to extract D_e^{app} from Supplementary Eq.3 D_e^{app} *(H2O/KCl) = 3.1 ± 1.3 * 10*$^{-10}$ *cm*^{2}*/s*

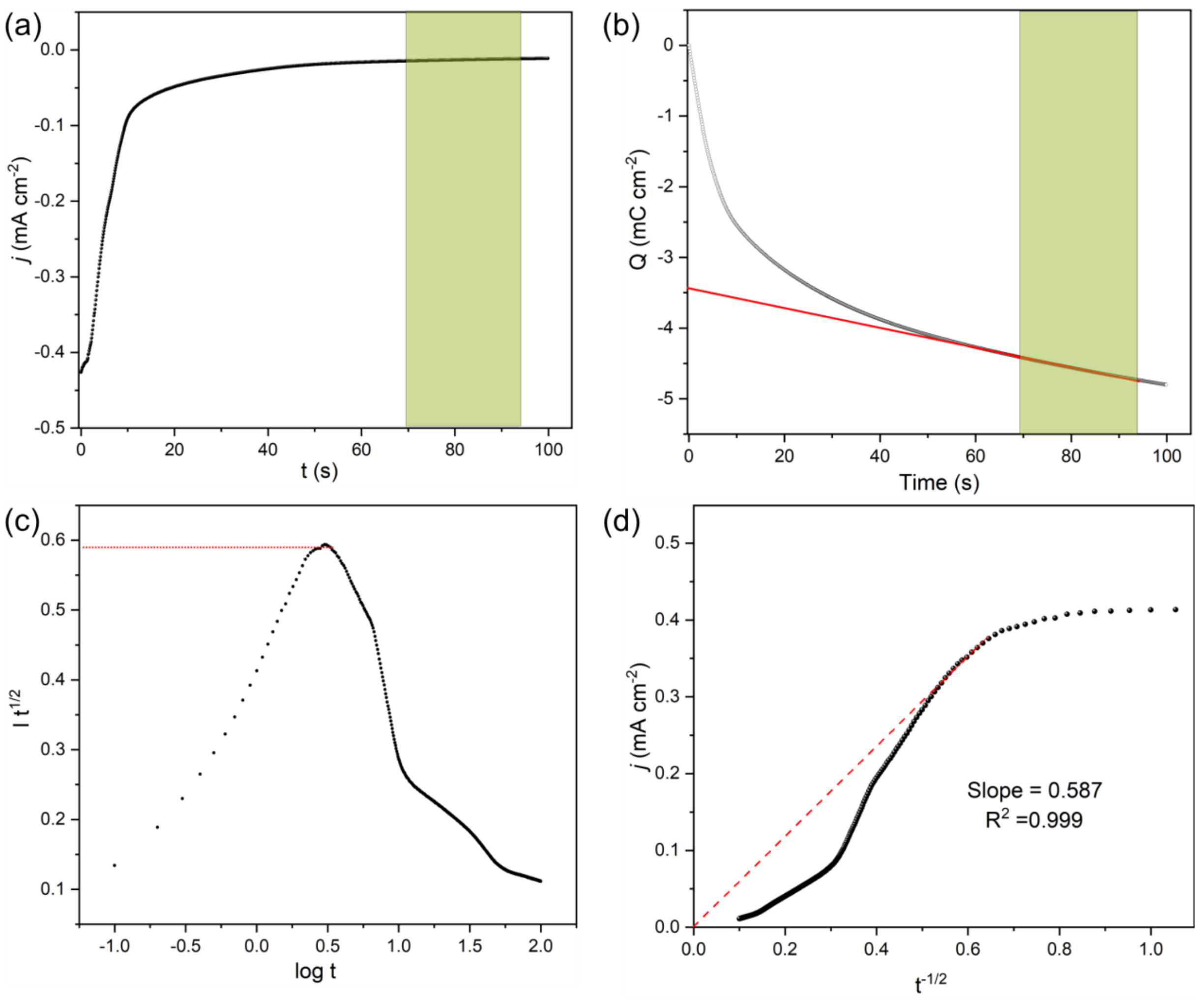

Figure S14. Electrochemical data for Zn-NDI|TiO_2|SC sample measured in 0.5 M $KClO_4$ in DMF. (a) Chronoamperometry (black circles) after stepping the potential to isolate the $NDI^{0/\bullet-}$ redox couple and (b) Chrono coulometry (black circles), the total charge passed after complete reduction of MOF film was determined after subtracting a residual background current (red solid line) Green boxes illustrate the window of time points chosen for linear fits for calculation of total surface concentration of redox active Unit. (According to Supplementary Eq.1) (c) Alternative representation of the Cottrell diffusion in the form of time dependence of the function $I(t)t^{1/2}$ calculated for the same data as in (figure S3, a), Dashed red horizontal lines define the maximum values of $I(t)t^{1/2}$ (*i.e.*, the Cottrell parameter) (d) Cottrell plot for Zn-NDI|TiO_2|SC measured in 0.5 M $KClO_4$ in DMF with a time step of 0.005 sec, After the potential step, the linear fit (red dashed line) used to extract D_e^{app} from Supplementary Eq.3 D_e^{app} (DMF/$KClO_4$) = 7.8 ± 2.2 * 10^{-11} cm^2/s

Photoelectrode stability

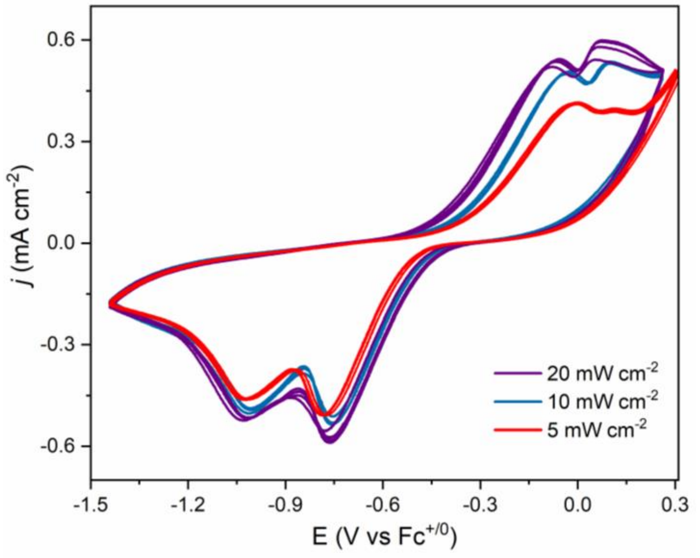

Figure S15. Consecutive CV scans of Zn-NDI|TiO_2|Si at three different illumination intensities. Shown are 5 scans for each light intensity, exhibiting negligible variations and demonstrating the stability of the photoelectrodes during the measurements.

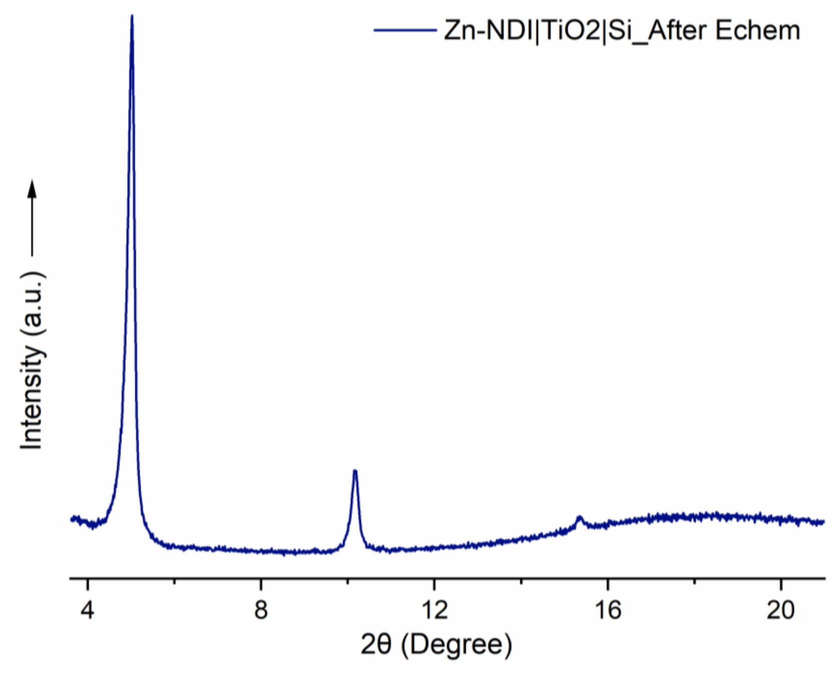

Figure S16 XRD of Zn-NDI|TiO_2|Si after photoelectrochemical experiments. The pattern is identical to that in the as-prepared sample, demonstrating the stability of the photoelectrodes during the photoelectrochemical measurements.

Electrochemically active surface area (EASA):

The **electrochemically active surface area** of the MOF modified electrode was calculated according to $\Gamma e = |Q| / (F\ S)$, where Γe is the electroactive linker concentration (mol cm^{-2}), Q is the charge (C), F is Faraday's constant (C mol^{-1}), and S is the surface area of the MOF-modified electrode (cm^2). This resulted in $\Gamma e = 3.83 \times 10^{-8}$ mol cm^{-2} as calculated from (Figure S15). With a film thickness of around 370 nm (calculated from SEM cross section), the total volumetric concentration of linker is approximately 1 M.

This value is then compared with the density of NDI linkers in the MOF per unit cell, obtained from the simulated crystal structure. This is equivalent molarity of a compound in crystalline form which is essentially molecular surface density (number of moles of formula unit per unit volume). Calculated unit cell volume 5216.24 $Å^3$, formula units per unit cell Z = 4 (Figure S1), N_A=6.022×1023 mol^{-1}, film thickness of around 370 nm (same as above), Surface area same as above 0.28 cm^{-2}. The calculation resulted in a molecular surface density is 4.71×10^{-8} mol cm^{-2} and the total volumetric concentration or molar density of linker is approximately 1.2 M.

Switching between limiting regimes in a different PIZOF MOF thin film on GaP

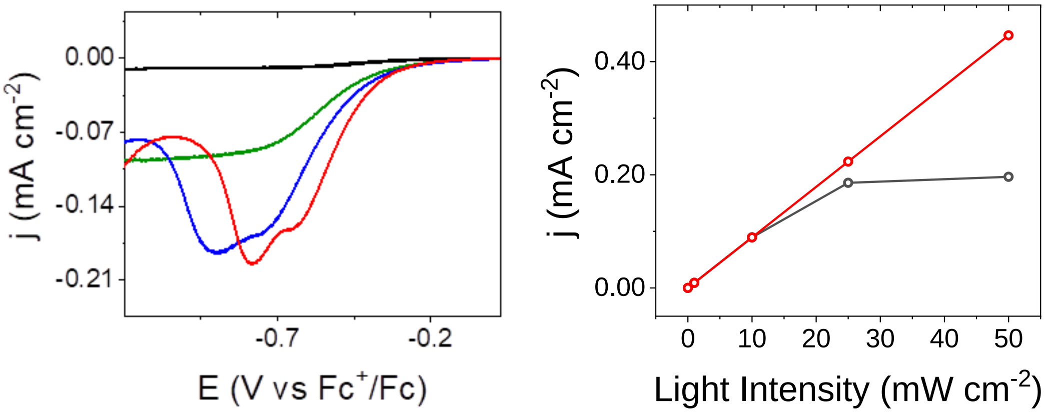

Figure S17. Cyclic voltammograms of the Zr(dcphOH-NDI) PIZOF MOF at GaP semiconductor under 0.5 sun (red), 0.25 sun (blue), 0.1 sun (green), and 0.01 sun (black) illumination at a scan rate of 100 mV s^{-1} in 0.5 M $LiClO_4$ in DMF (left). Maximum current densities as a function of light intensity (blue trace), and its deviation from linearity (red trace), showing a switch in limiting phenomenon similar to those in the Zn-NDI systems in the main manuscript (right).

Reference

(1) Beiler, A. M.; McCarthy, B. D.; Johnson, B. A.; Ott, S. Enhancing photovoltages at p-type semiconductors through a redox-active metal-organic framework surface coating. *Nat. Commun.* **2020**, *11* (1), 5819.
(2) Kumar, A.; Li, J.; Inge, A. K.; Ott, S. Electrochromism in Isoreticular Metal–Organic Framework Thin Films with Record High Coloration Efficiency. *ACS Nano* **2023**, *17* (21), 21595-21603.
(3) Wade, C. R.; Corrales-Sanchez, T.; Narayan, T. C.; Dincă, M. Postsynthetic tuning of hydrophilicity in pyrazolate MOFs to modulate water adsorption properties. *Energy Environ. Sci.* **2013**, *6* (7), 2172-2177.
(4) Kim, D. J.; Jung, Y. H.; Bharathi, K. K.; Je, S. H.; Kim, D. K.; Coskun, A.; Choi, J. W. An Aqueous Sodium Ion Hybrid Battery Incorporating an Organic Compound and a Prussian Blue Derivative. *Advanced Energy Materials* **2014**, *4* (12), 1400133.
(5) Albanese, E.; Civalleri, B.; Ferrabone, M.; Bonino, F.; Galli, S.; Maspero, A.; Pettinari, C. Theoretical and experimental characterization of pyrazolato-based Ni(ii) metal–organic frameworks. *J. Mater. Chem.* **2012**, *22* (42), 22592-22602.
(6) Myers, D. R.; Kurtz, S. R.; Emery, K.; Whitaker, C.; Townsend, T. Outdoor meteorological broadband and spectral conditions for evaluating photovoltaic modules. In *Conference Record of the Twenty-Eighth IEEE Photovoltaic Specialists Conference - 2000 (Cat. No.00CH37036)*, 15-22 Sept. 2000, 2000; pp 1202-1205.
(7) Levi, M. D.; Demadrille, R.; Pron, A.; Vorotyntsev, M. A.; Gofer, Y.; Aurbach, D. Application of a Novel Refinement Method for Accurate Determination of Chemical Diffusion Coefficients in Electroactive Materials by Potential Step Technique. *J. Electrochem. Soc.* **2005**, *152* (2), E61.
(8) Levi, M. D.; Gofer, Y.; Cherkinsky, M.; Birsa, M. L.; Aurbach, D.; Berlin, A. Electroanalytical features of non-uniformly doped conducting poly-3-(3,4,5-trifluorophenyl)thiophene films. *Physical Chemistry Chemical Physics* **2003**, *5* (13), 2886-2893.
